# Identification of small molecule compounds targeting the interaction of HIV-1 Vif and human APOBEC3G by virtual screening and biological evaluation

**DOI:** 10.1038/s41598-018-26318-3

**Published:** 2018-05-23

**Authors:** Ling Ma, Zhixin Zhang, Zhenlong Liu, Qinghua Pan, Jing Wang, Xiaoyu Li, Fei Guo, Chen Liang, Laixing Hu, Jinming Zhou, Shan Cen

**Affiliations:** 1 Institute of Medicinal Biotechnology, Chinese Academy of Medical Sciences & Peking Union Medical College, Beijing, China; 2 Lady Davis Institute for Medical Research, Jewish General Hospital, McGill University, Montreal, QC Canada; 3 Institute of Pathogen Biology, Chinese Academy of Medical Sciences & Peking Union Medical College, Beijing, China

## Abstract

Human APOBEC3G (hA3G) is a restriction factor that inhibits human immunodeficiency 1 virus (HIV-1) replication. The virally encoded protein Vif binds to hA3G and induces its degradation, thereby counteracting the antiviral activity of hA3G. Vif-mediated hA3G degradation clearly represents a potential target for anti-HIV drug development. Herein, we have performed virtual screening to discover small molecule inhibitors that target the binding interface of the Vif/hA3G complex. Subsequent biochemical studies have led to the identification of a small molecule inhibitor, IMB-301 that binds to hA3G, interrupts the hA3G-Vif interaction and inhibits Vif-mediated degradation of hA3G. As a result, IMB-301 strongly inhibits HIV-1 replication in a hA3G-dependent manner. Our study further demonstrates the feasibility of inhibiting HIV replication by abrogating the Vif-hA3G interaction with small molecules.

## Introduction

Human Apolipoprotein B mRNA-editing catalytic polypeptide-like 3 G (hA3G), which belongs to the APOBEC superfamily containing at least 10 members, is a restriction factor that inhibits the replication of HIV-1^1^. Virion-associated hA3G is able to convert cytosine to uracil in newly synthesized viral DNA, thus causing hypermutation and inactivation of the new viral genome^2–4^. Furthermore, several lines of evidence indicate that hA3G also impairs viral DNA synthesis and integration through an antiviral mechanism distinct from deamination^5,6^. As a countermeasure, HIV-1 Vif efficiently downregulates hA3G through binding to hA3G. Through recruiting an ubiquitin ligase complex containing cullin-5(CUL5), Vif causes polyubiquitination and proteasomal degradation of hA3G, thus preventing hA3G from incorporation into HIV-1 particles and overcoming the anti-HIV-1 activity of hA3G^7–10^.

hA3G contains two tandem CD domains, the N-terminal (CD1) and the C-terminal (CD2). The CD2 domain exhibits deaminase activity, whereas the CD1 domain is catalytically inactive. Regardless, mutations in the CD1 domain affect multiple aspects of hA3G function, including dimerization, virion incorporation, subcellular distribution and interaction with Vif. The interaction between hA3G and Vif plays a critical role in Vif mediated hA3G degradation. Previous studies have suggested that the β4-α4 loop of hA3G, comprising amino acids 122–130, is involved in the hA3G-Vif interaction and is required for the subsequent degradation by Vif^11–15^. The residues D128 and P129 are crucial for the binding of Vif to hA3G. For example, the D128K mutant of hA3G is completely resistant to Vif, while mutating HIV-1 Vif motif 14-DRMR-17 to 14-SEMQ-17 enables the mutated Vif protein to degrade the hA3G-D128K mutant^14–17^, suggesting an important interaction between Vif-14–17 and hA3G-128. In addition, Gooch and Cullen have reported that the replacement of 124-YYFW-127 in hA3G with the corresponding hA3A sequence abolishes the sensitivity of hA3G to Vif. A reciprocal mutation in hA3A changes hA3A into a target for Vif-mediated degradation^18^. This suggests that the 122-RLYYFWDP-129 motif in hA3G dictates its degradation by Vif. In agreement with this observation, our molecular modeling data predict an interaction of Vif with the central residues (124-YYFW-127)^19^. A recent study revealed that hA3G-125 interacts with amino acids 19 and 22 of Vif, and that hA3G-130 interacts with amino acid 82 of Vif^20^. These results collectively demonstrate the key role of hA3G β4-α4 loop in Vif binding and Vif-mediated degradation.

Disrupting the interaction of hA3G and Vif, thus inhibiting Vif-mediated degradation of hA3G, represents a new anti-HIV-1 strategy. Indeed, several groups have screened for molecules that can inhibit Vif-mediated hA3G degradation using function-based assays, and have reported lead compounds including RN-18^21,22^, IMB-26/35^23^, MM-1/2^24^, and VEC-5^25^. Molecular modeling has been frequently used to investigate the action mechanism of these active compounds and to explore the novel compound structure that may inhibit the Vif-mediated hA3G degradation. For examples, RN-18 has been proposed to bind Vif, and molecular docking has predicted the binding mode of RN-18 with Vif^22^. Through virtual screening and biochemical assay, VEC-5 has been identified as the inhibitor of Vif via direct binding to the ELOC protein^25^. Recently, a compound named ZBMA-1 was identified as a potent HIV-1 replication inhibitor through protecting hA3G protein. Further studies of the co-immunoprecipitation and molecular docking have indicated that ZBMA-1 inhibits the Vif-mediated hA3G degradation via affecting the binding of Elongin C with Vif protein^26^.

Therefore, molecular modeling provides a powerful tool in the drug discovery of targeting Vif-mediated degradation of hA3G. Our previous work has shown that IMB-26/25 blocks Vif-mediated hA3G degradation through binding with hA3G. Further molecular docking study suggests that IMB-26/35 binds to a putative site near the 124-YYFW-127 motif in the hA3G-CD1 model^27^, which has been generated through homology modeling based on the template APO2 dimer structure (PDBID: 3IQS)^28^. This putative site provides a potential target for virtual screening. Importantly, the recently solved human APOBEC3F (hA3F) structure (PDB ID, 4J4J)^29^ provides a more suitable template than the APO2 protein. Herein, we have established the hA3G model based on the hA3F structure through homology modeling. This hA3G model has allowed us to identify a small molecular inhibitor IMB-301 via virtual screening. Further biochemical experiments have verified that IMB-301 binds to hA3G, restores hA3G expression in the presence of Vif, and inhibits the replication of HIV-1 in a hA3G-dependent manner.

## Results

### Virtual screening of small molecule compounds that target the interface of hA3G/Vif interaction

**Figure 1:**
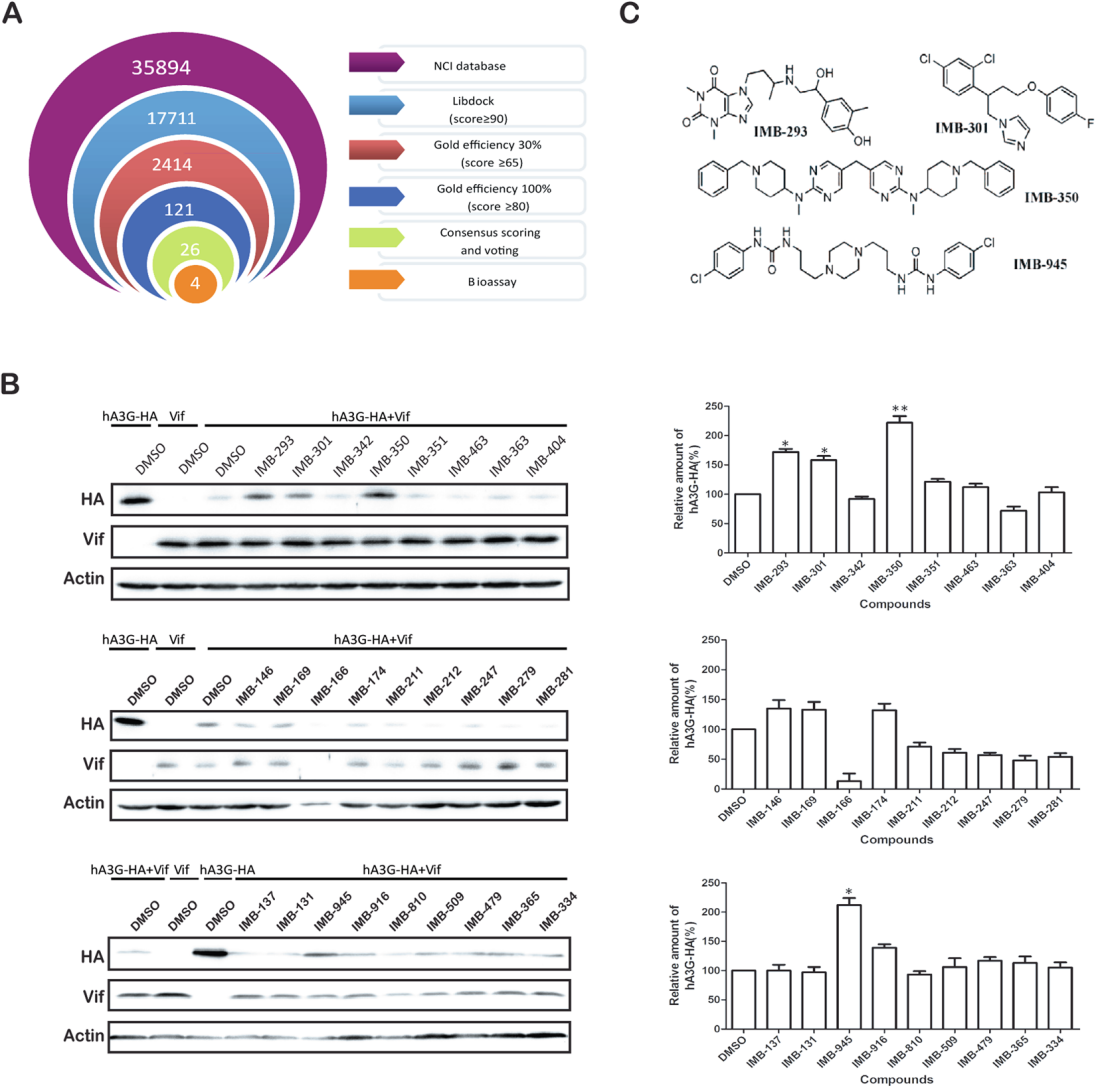
Virtual screening of small molecule compounds targeting the interface of hA3G/Vif interaction. (**A**) Virtual screening protocol used for the discovery of potential hA3G binders. The numbers indicate the number of compounds that were obtained for each screening step. (**B**) 293 T cells were co-transfected with hA3G-HA and Vif. Twelve hours post transfection, compounds(10 μM) were added. Forty-eight hours post transfection, cell lysates were harvested and proteins were detected by WB (left panel). Total amounts of DNA were maintained the same between transfections by supplementation of empty vector DNA. The relative hA3G-HA and β-actin levels in Fig. 1B were quantified by Image J software and normalized with the corresponding DMSO control group of each blot (right panel). The data from three independent experiments are summarized in the bar graph and represent the mean ± s.e.m (err bar). The value of controls is arbitrarily set as 100 (%). Asterisk (*) indicate P < 0.05 and double asterisks (**) indicate P < 0.01 relative to controls (the GraphPad Prism software). (**C**) Structures of IMB-293 (NSC 293893), IMB-301 (NSC 301209), IMB-350 (NSC 350004), IMB-945 (NSC 94511).

We first established a homology model of hA3G based on the crystal structures of hA3F (PDB ID, 4J4J) and the hA3G CD2 domain (PDB ID 3V4K). The results of sequence alignment showed that the template hA3F and the CD1 domain of hA3G have a high sequence identity of 41.5%, with a sequence similarity of 57.9%, which indicates that the template is suitable for homology model building. The Verify Score of the hA3G structure (149.35) is close to the Verify Expected High Score (173.24), suggesting the high quality of the hA3G structure model. Then we predicted the binding sites for the hA3G model using the site finder module implemented in Molecular Operating Environment (MOE, Chemical Computing Group Inc., Montreal, QC, Canada)^30^. As a result, a predicted binding site was found close to the reported Vif-hA3G interface (motif 122–132), which is similar to the previous putative site and involves residues Arg14, Asp15, Phe17, Ser18, Try19, Phe21, Tyr22, Arg24, Ser93, TRP94, Ala121, Arg122, Phe157, Trp175, Asn177, Leu178, and Tyr181. The schedule of virtual screen was shown in Fig. 1A. The NCI open compound database (dtp.cancer.gov) was selected as the ligand database for our virtual screen. First, the database was selected according to the drug-like properties of the compound using “Prepare Ligands” module in Discovery Studio 2.5 (Accelrys, San Diego, California, USA) and further selected through the OIDD package (Lilly’s Open Innovation Drug Discovery initiative)^31^. 35894 entries were left. The optimization database was screened against the predicted binding site on hA3G using docking module Libdock in DS^32^, and was further evaluated using docking program GOLD (The Cambridge Crystallographic Data Centre, Cambridge, UK)^33^. Finally, after visual inspection, 26 hits were selected and obtained from NCI for further assessment.

To evaluate the biological activity of the 26 hits, we assessed their effect on hA3G expression in the presence of HIV-1 Vif. 293 T cells were co-transfected with the expression vectors for hA3G-HA and Vif, and then treated with 10 μM of each compound. The results in Fig. 1B showed that four compounds, IMB-293, IMB-301, IMB-350 and IMB-945, restored hA3G expression in the presence of Vif, compared with that in the cells treated with DMSO. This suggests that the four compounds are able to inhibit the degradation of hA3G by HIV-1 Vif.

### Small molecules restore hA3G expression in the presence of Vif through disrupting Vif-hA3G interaction

**Figure 2:**
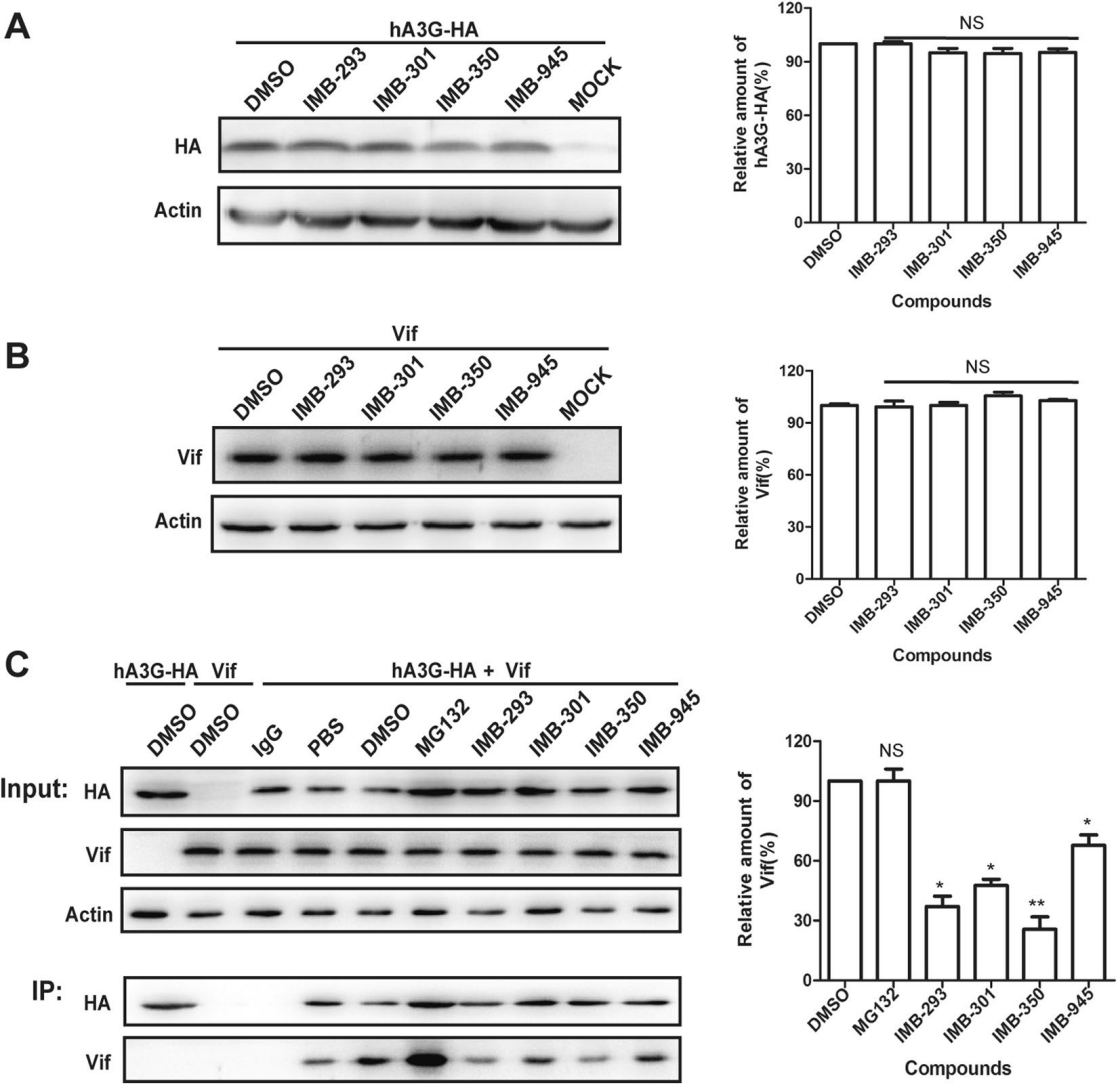
Small molecules restore hA3G expression in the presence of Vif through disruption of the Vif-hA3G interaction. 293 T cells were transfected with hA3G-HA (**A**) and Vif. (**B**), respectively, and treated with the compounds 12 hours post transfection, followed by WB analysis of cell lysate. (**C**) 293 T cells were co-transfected with hA3G-HA and Vif, followed by the treatment with 10μMof each compound. Equal amounts of cell lysates and anti-HA immunoprecipitated materials were analyzed by WB using anti-HA and anti-Vif antibody, respectively. Normal rabbit IgG was used as a nonspecific IgG control in immunoprecipitation. Total amounts of DNA were maintained the same between transfections by supplementation of empty vector DNA. Left panel represents the relative hA3G-HA (**A**) and Vif (**B** and **C**) expression levels quantified by Image J software and normalized with the DMSO control group. In immunoprecipitation assay for quantitatively measuring hA3G/Vif interaction (**C**), the amounts of Vif were firstly normalized with that of hA3G-HA in anti-HA immunoprecipitates. The data from three independent experiments are summarized in the bar graph and represent the mean ± s.e.m (err bar). The value of controls is arbitrarily set as 100%. Asterisk (*) indicate P < 0.05 and double asterisks (**) indicate P < 0.01 relative to controls (the GraphPad Prism software). NS, not statistically significant (P > 0.05).

We next investigated whether the four compounds interrupt the Vif/hA3G interaction and thus inhibit Vif-mediated degradation of hA3G. These compounds increased hA3G-HA level in the presence of Vif by approximate 0.85–2.5 fold compared with that of control group (Fig. 1C); whereas they exhibited no effect on the expression of hA3G-HA in the absence of Vif (Fig. 2A) as well as Vif alone (Fig. 2B). These data suggest that the increased hA3G-HA expression following treatment with these compounds results from protecting hA3G-HA from the Vif-mediated proteasomal degradation, rather than stimulating hA3G-HA expression or reducing Vif level. This conclusion is further corroborated by the fact that the four compounds significantly reduced the amount of Vif that was co-immunoprecipitated with hA3G-HA (Fig. 2C), demonstrating that they inhibit the Vif-hA3G interaction and thereby block hA3G degradation by Vif.

### IMB-301 inhibits the replication of HIV-1 in the presence of hA3G

**Figure 3:**
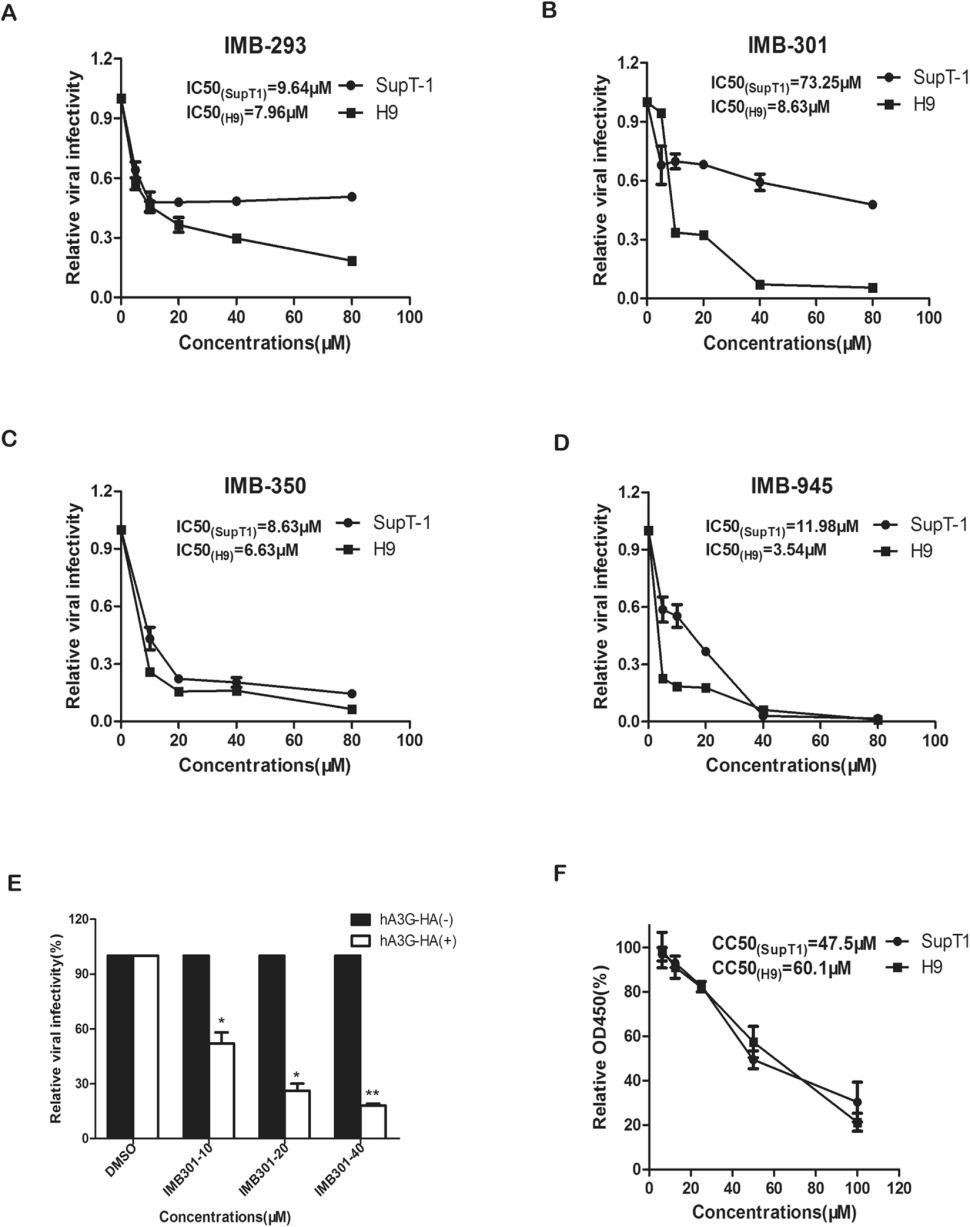
IMB-301 inhibits the replication of HIV-1 in the presence of hA3G. H9 and SupT1 cells were infected with wild-type HIV-1(NL4-3) in the presence of IMB-293 (**A**), IMB-301 (**B**), IMB-350 (**C**), or IMB-945 (**D**) at various concentrations. The infectivity of nascent viruses in the supernatant was measured by infecting the TZM-bl indicator cells. (**E**) 293 T cells were co-transfected with pNL4-3Luc(R-E-), VSVG and hA3G-HA (or pcDNA3.1), followed by the treatment with various concentration of IMB-301. The supernatants were collected and then used to infect the SupT1 cells. (**F**) H9 or SupT1cells were incubated with various concentrations of IMB-301 or DMSO for 48 hours, and cytotoxic effects were then measured using the CCK8 assays. Equal volumes of DMSO were added into the culture medium, which contain different concentrations of the compounds, in order to keep constant final DMSO concentration as 1% (v/v). The data from three independent experiments are summarized in the bar graph and represent the mean ± s.e.m (err bar). The value of controls is arbitrarily set as 1 (**A**–**D**) or 100% (**E** and **F**). Asterisk (*) indicate P < 0.05 and double asterisks (**) indicate P < 0.01 relative to controls (the GraphPad Prism software).

We next asked whether these small molecules are able to inhibit HIV-1 replication in ahA3G-dependent manner. Previous studies have shown that the level of endogenous hA3G varies in human T-cell lines^1^, i.e., higher level in H9 but much lower level in SupT1. Higher anti-HIV activity of the above compound is therefore expected in H9 than SupT1 cells if this compound acts as an inhibitor against hA3G degradation by Vif. To test this, H9 and SupT1 cells were infected with wild-type HIV-1(NL4-3) in the presence of the above compounds at various concentrations. The infectivity of nascent viruses in the supernatant was measured using TZM-bl indicator cell. In consistence with previous reports, we found that the hA3GmRNA level in SupT1 cells was approximate 10% of that in H9 cells (Figure S1A), and the hA3G protein level in SupT1 was significantly less than that in H9 (Figure S1B). As shown in Fig. 3B, IMB-301 exhibited much higher potency against HIV-1 in H9 cells (IC50 = 8.63 μM) than in SupT1 cells (IC50 = 73.25 μM), with an 8.5-fold difference in the IC50 values, whereas no significant difference in anti-HIV activity were observed with the other three compounds (Fig. 3A,C and D). This suggests that the majority of anti-HIV activity of IMB-301 depends on the presence of hA3G. In agreement with this observation, IMB-301 significantly inhibited the infectivity of VSVG pseudotyped HIV-1 that was produced from hA3G-HA-expressing 293 T cells in a dose-dependent manner compared with that produced from control 293 T cells (Fig. 3E). We next examined the cytotoxicity of IMB-301. H9 or SupT1cells were incubated with various concentrations of IMB-301 for 48 hours, and cell viability was then measured with the CCK8 assay. IMB-301 showed similar cytotoxic profiles in H9 and SupT1 cells, and no obvious toxicity was observed in H9 cells at the concentration of its anti-HIV-1 IC50 value (8.63 μM) (Fig. 3F). Taken together, these results suggest that IMB-301 inhibits HIV-1 replication through protecting hA3G from Vif-mediated degradation.

### IMB-301 increases virion incorporation of hA3G

**Figure 4:**
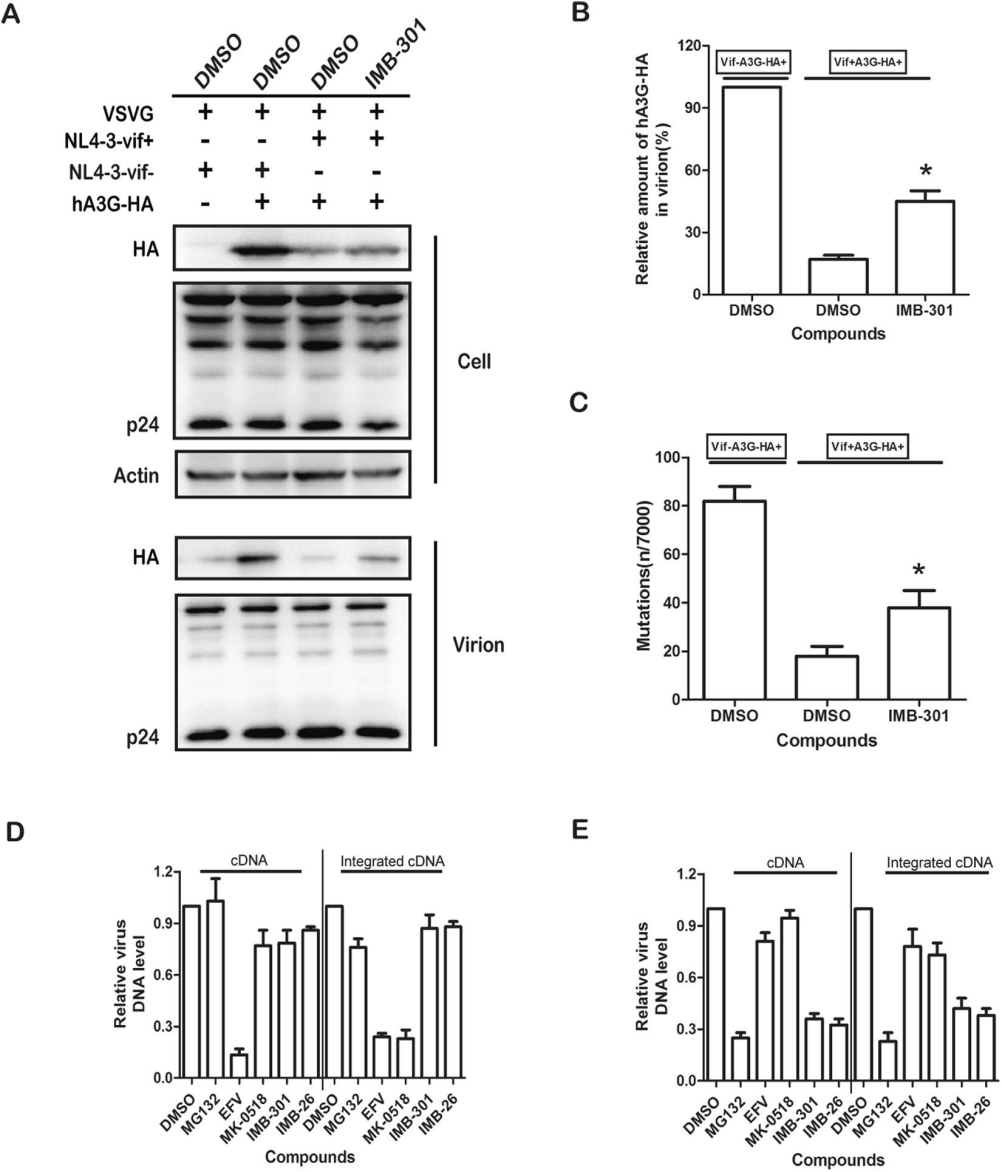
The small molecules increase hA3G incorporation into Vif-proficient virus particles. 293 T cells were cotransfected with VSVG, pNL4-3(R-E-) (or pNL4-3-Vif) and hA3G-HA (or pcDNA 3.1) plasmid DNA, followed by the treatment with 10 µM of IMB-301. (**A**) the cells and virion lysates were analyzed by WB. (**B**) Bands in WB were quantitated using Image J software. (**C**) The resultant viruses were used to infect TZM-bl cells. Total DNA in the infected cells was isolated and PCR amplified. The PCR products were sequenced to identify the G to A mutations. (**D**) The effect of IMB-301 on HIV-1 infectivity in 293 T cells expressing hA3G. (**E**) The infectivity of nascent viruses from IMB-301 treated 293 T cells expressing hA3G. (**D**) and(**E**) Two groups of 293 T cells were prepared as described in the methods. U5-gag, Alu-LTR and IN represents plus strand DNA, transfer DNA and integrated proviral cDNA. Four pairs of primers were used. qPCR result was normalized using the GAPDH DNA levels. The data from three independent experiments are summarized in the bar graph and represent the mean ± s.e.m (err bar). The value of controls is arbitrarily set as 100% (**B**) or 1 (**D** and **E**). Asterisk (*) indicate P < 0.05 relative to controls (the GraphPad Prism software).

Viral incorporation of hA3G into HIV-1 virion is a prerequisite for its anti-HIV-1 action. We therefore investigated if the inhibition by IMB-301 of HIV-1 replication is a result of an increase in virion encapsidation of hA3G-HA in the presence of Vif. 293 T cells were co-transfected with HIV-1 proviral DNA and hA3G-HA expressing plasmid, followed by the treatment with this compound. The resultant viral particles in the culture supernatant were collected. Western blot of viral lysate revealed that treatment with IMB-301 significantly increased the amount of hA3G-HA in the virions (Fig. 4A,B). This suggests that IMB-301 is able to increase virion encapsidation of hA3G via its ability to impair the degradation of hA3Gby Vif, which results in hA3G-dependent anti-HIV activity.

As a hallmark of hA3G’s cytidine deaminase activity, the virion-associated hA3G causes G to A hypermutation in viral genome during the new round of infection. To functionally validate the result of virion encapsidation of hA3G (Fig. 4A,B), we further measured dG to A hypermutations in the cells that were infected with the resultant viruses prepared in above experiments. The proviral DNA was extracted from the infected cells and amplified with PCR for the viral DNA fragment spanning nef, U3 and R regions as described previously^34,35^. A significant increase in the number of G to A mutation was observed in IMB-301 treated group (Fig. 4C), and the hypermutation rate gradually elevated concurrent with the increasing concentrations of IMB-301 used (Figure S2).

Virion-associated hA3G strongly inhibits HIV replication during the new round of infection, while cellular hA3G has no effect on newly infected HIV-1. Therefore, if IMB-301 targets Vif-mediated hA3G degradation, we conceived that treating hA3G-HA-containing cells with the compound would impair the infectivity of viruses produced from these cells, but have no effect on the replication of viruses infecting these cells. To the end, we monitored HIV-1 infection by performing real-time PCR to quantify the products of HIV-1 reverse transcription as well as viral DNA integration in the infected 293 T cells. Efavirenz (EFV, a NNRTI) and Raltegravir (RAL, an INSTI) were used as controls. As shown in Fig. 4D, in hA3G-HA-expressing 293 T cells infected with HIV-1, the treatment with EFV or RAL significantly inhibited HIV-1 reverse transcription and integration, respectively. However, IMB-301 had no effect on either viral DNA production or the level of integrated viral DNA, suggesting that IMB-301 does not inhibit the replication of incoming viruses (Fig. 4D). Next, hA3G-HA expressing 293 T cells were transfected with HIV-1 proviral DNA to produce viruses, followed by the treatment with these compounds. Then the same amount of the resultant viruses were used to infect 293 T cells, HIV-1 reverse transcription and integration were examined as described above (Fig. 4E). In contrast to what we have observed in Fig. 4D, treating virus-producing cells with IMB-301 caused significant reduction in the infectivity of the resultant viruses, i.e., less viral cDNA and integrated DNA, whereas no inhibitory effect was observed for EFV and RAL (Fig. 4E). This suggests that IMB-301 is capable of impairing the infectivity of progeny viruses rather than incoming viruses, which has also been observed for IMB-26 that was reported previously to inhibit hA3G degradation by Vif^36^. These results further demonstrate that the anti-HIV activity of IMB-301 results from its ability to inhibit Vif-mediated hA3G degradation, leading to an increase in viral incorporation of hA3G.

### IMB-301 is unable to inhibit hA3F degradation by Vif

**Figure 5:**
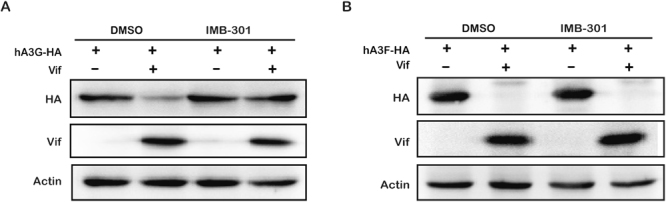
IMB-301 specifically inhibitshA3G but not hA3F degradation. 293 T cells were co-transfected with Vif and eitherhA3G-HA (**A**) or hA3F-HA (**B**), followed by the treatment of IMB-301. Then cell lysates were analyzed by WB. Total amounts of DNA were maintained the same between transfections by supplementation of empty vector DNA.

hA3F is also a member of theAPOBEC3 family, potently inhibits Vif-deficient HIV-1 replication, and HIV-1 Vif is able to bind to hA3F and counteracts its antiviral activity via proteasome-dependent degradation^3,37^. Although hA3F is highly homologous to hA3G, hA3F shows unique characteristics that are different from hA3G in term of the residues that are involved in binding to Vif^13,37^. It is therefore interesting to study whether IMB-301 also inhibits Vif-mediated degradation of hA3F. 293 T cells were therefore co-transfected with plasmids expressing Vif and either hA3G-HA or hA3F-HA, followed by the treatment with IMB-301. Western blot of cell lysate showed that this compound efficiently restored the level of hA3G (Fig. 5A) but not that of hA3F (Fig. 5B) in the presence of Vif, which suggests that IMB-301 is a specific inhibitor for Vif-mediated hA3G degradation and does not exert a general inhibitory effect on the proteasomal pathway.

### IMB-301 directly binds to hA3G

**Figure 6:**
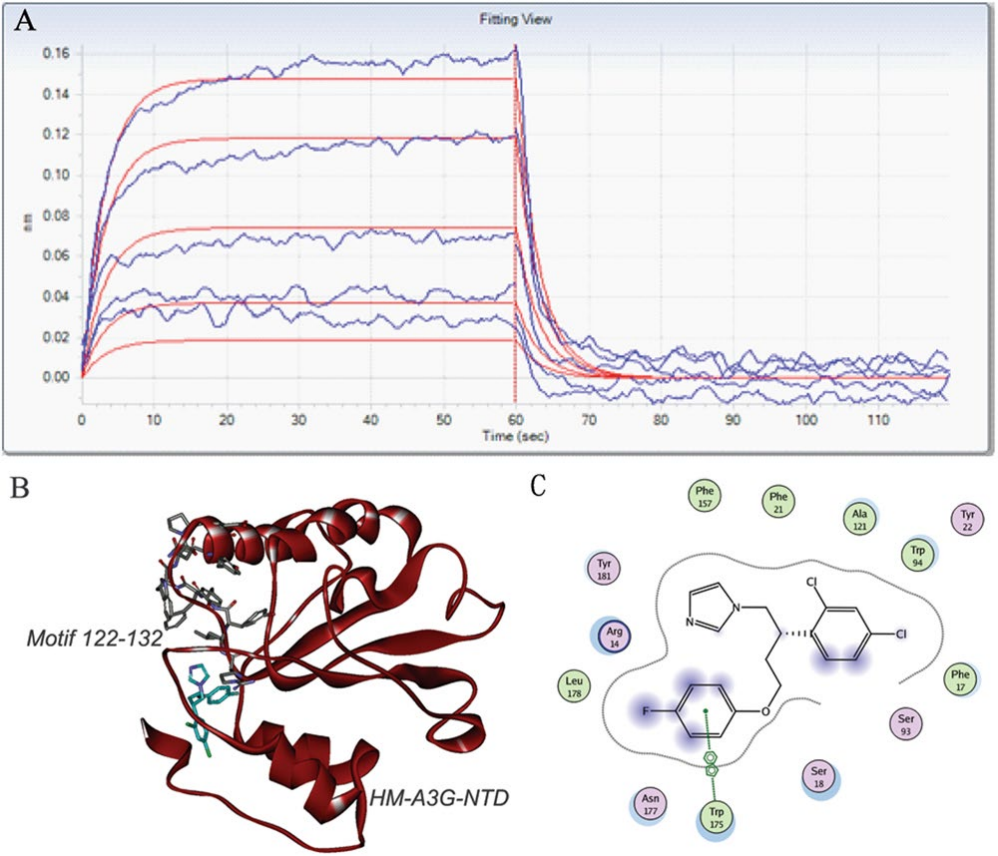
IMB-301 binds to hA3G. (**A**) Octet binding of IMB-301 to hA3G was performed as described in Materials and Methods. (**B**) The predicted binding mode of IMB-301 in the binding site for the homology structure of hA3G-NTD (HM-hA3G-NTD). (**C**) The detailed interactions between IMB-301 and hA3G-NTD.

To test whether IMB-301 binds to hA3G directly, we performed the Octet binding assay using biotinylated hA3G on super streptavidin sensors and IMB-301 (Fig. 6A). The kinetics constants are shown in Table 1. IMB-301 had a dissociation equilibrium constant of 15.6 μM. IMB-301 directly bound hA3G at significantly higher levels than the other compounds (Figure S3). These data suggest that IMB-301 directly binds to hA3G. We further investigated the binding mode of IMB-301 with hA3G. IMB-301 was found at the location close to motif 122–132 (Fig. 6B), and formed hydrophobic interactions with Phe17, Phe21, Trp94, Ala121, Phe157, Trp175, and Leu178 as well as π-π interaction with Trp175 at the binding site (Fig. 6C). Importantly, in addition to motif 122–132, Trp94, Phe157, and Trp175 were also found playing important roles in Vif and hA3G interaction^13^. These results suggest that IMB-301 disrupts the interaction between Vif and hA3G through binding to the site proximal to motif 122–132.

**Table 1:** Kinetics of IMB-301 binding to hA3G in the ForteBio’s Octet System.

Analyte	Ligand (hA3G)				
	K_on_ (1/Ms)	K_on_ Error	K_off_ (1/s)	K_off_Error	K_D_ (M)
IMB-301	2.01E + 04	5.94E + 04	3.13E − 01	9.85E − 03	1.56E − 05

^*^The kinetic constants were calculated using the ForteBio’s Octet System for the interaction between IMB-301 and hA3G. K_on_, association rate constant; K_off_, dissociation rate constant; K_D_, dissociation equilibrium constant.

## Discussion

Disrupting Vif-mediated hA3G degradation represents a promising anti-HIV-1 strategy. Until recently, IMB-26/35 as well as several other small molecules has been reported to block Vif-mediated hA3G degradation. Zinc chelate TPEN was shown to impair the ability of Vif to degrade hA3G, but the essential role for zinc in cells limits the further development of TPEN as an anti-HIV drug. The small molecule compound RN-18 can specifically degrade Vif in the presence of hA3G^21^. Through selectively degrading Vif in the Vif/hA3G complex, it blocks the Vif-mediated degradation of hA3G and strongly inhibits the replication of HIV-1. However, the precise mechanism remains unclear. Yu *et al*. reported a small molecule, VEC-5, that targets the interface of Vif and ELOC, thus inhibits Vif-mediated degradation of hA3G and replication of HIV-1 in hA3G-positive cells^22,25^. However, since the BC-box is highly conserved and is present in many important cellular proteins, a compound specifically targeting the BC-box of Vif is very difficult to attain. Targeting the interface between Vif/hA3G represents the most attractive strategy to block Vif-mediated degradation of hA3G. Recently, through high-throughput screening, several compounds such as MM-1/2^24^ and N41^38^ were identified as competitive inhibitors of Vif and hA3G interaction. However, the precise binding sites of these compounds on either Vif or hA3G are unknown. Herein, we have constructed a model of hA3G, and predicted a binding site close to the Vif/hA3G interface. Also we have found an active compound IMB-301 that disrupts the interaction of Vif/hA3G via the virtual screening against the predicted site. This site could also be the binding position of other reported compounds such as MM-1/2^24^, N41^38^, and IMB-26/35^23^.

The structural information is critical in rational drug design. When we prepared this manuscript, the NMR structure of hA3G-NTD (PDB ID, 2MZZ), which shares 80% sequence identity with original hA3G, was solved^39^. We therefore aligned our model to the experimental hA3G-NTD structure, and the superimposition of the two structures gives a RMSD value of 4.21 Å for overall structure (Figure S4).In addition, these two structures bear similar folds, which indicate that our model is quite reliable. Importantly, a predicted binding site is also present in the NMR structure of hA3G-NTD, which is at the similar location to the site we have used for virtual screen (Figure S5A). Further docking of IMB-301 to the binding site also gave the similar binding mode (Figure S5B). Besides the structure information of hA3G-NTD, the crystal structure of Vif (PDB ID, 4N9F)^40^, which bound to CBF-β and CUL5 E3 ligase complex, was recently solved. This latter structure will help to understand how Vif interacts with hA3G and also provide structure information for rational design of Vif/hA3G blockers.

When we attempted to predict the binding sites in the hA3F structure using the site finder module, the identified site in the hA3G model which was used in virtual screening could not be found at the similar location in the hA3F structure. It has also been reported that the interface of Vif (residues 17, 76, 171, 173)^41^ and hA3F (motif 289–294, residue 324)^42,43^ interaction is entirely different from that of Vif (residues 19, 22, 82) and hA3G (motif 122–130)^18^. Therefore, the compounds identified in this work are able to inhibit the interaction between hA3G and Vif (Fig. 2) and Vif-induced degradation of hA3G (Fig. 1), but exerted no effect on hA3F degradation by Vif (Fig. 5 and Figure S6). This further supports the reliability of the homology model. It is worth noting that this selection process cannot exclude other possible activities of the hits that impair HIV-1 replication, although the docking screening provides relatively accurate prediction on the binding of small molecules to hA3G. Therefore, it is possible that hA3G-independent activity of the hits other than IMB-301 contributes to the majority of their anti-HIV effect. This might explain why IMB-293 and IMB-945, both of which are able to inhibit Vif-mediated degradation of hA3G, show anti-HIV activity in a hA3G-independent manner in H9 and SupT1 cells.

Similarly, in addition to binding to hA3G, we currently cannot exclude the possibility that the compounds may also bind to other proteins like Vif or ELOC to inhibit the Vif-mediated hA3G degradation. However, due to the poor solubility and stability of Vif protein^44,45^, we are unable to purify the Vif protein for the Octet binding assay. In addition, recent studies showed that the interaction with human protein CBF-β involves the solubility, and more importantly, the functional conformation of HIV-1 Vif. Therefore, it might be more feasible to investigate the binding of the compound to the Vif-CBF-β complex in the future. Several lines of evidence suggest that the same E3 ubiquitin ligase complexes are involved in the Vif-induced degradation of both hA3G and hA3F. Since IMB-301 is able to block the degradation of hA3G but not hA3F, the compound does not disrupt the formation of E3 ligase complex. Therefore, it is unlikely that the compound binds to proteins that are involved in degradation of both hA3G and hA3F. Nevertheless, we could not exclude the possibility that IMB-301 might bind to some unknown proteins that are required for the degradation of hA3G but not hA3F. Particularly, the Tanimoto similarity values between IMB-301 and other active compounds such as IMB-26, MM-1, RN-18 (Table S1), which were calculated through Open Babel^46^ are all below 0.25 (Table S1), indicates that IMB-301 represents a lead compound with a novel core-structure for further optimization.

## Conclusions

Taken together, we have identified a small molecular inhibitor IMB-301 via virtual screening according to the hA3G model. Further biochemical experiments have shown that IMB-301 binds to hA3G, restores hA3G expression in the presence of Vif, and inhibits the replication of HIV-1 in a hA3G-dependent manner. Our results demonstrate the possibility of inhibiting HIV replication by abrogating the Vif-hA3G interaction with small molecules.

## Methods and Materials

### Structure-based molecular docking of NCI database small molecule compounds

The hA3G sequence (residues 1–384, Uniprot Entry: Q9HC16, www.uniprot.org) was defined as target sequence. The crystallized hA3F (PDB ID, 4J4J, www.pdb.org)^29^ and the crystallized hA3G CD2 domain structure (PDB ID 3V4K)^47^ served as the template of the NTD and CTD domains, respectively. The hA3G structure model was further built in discover studio (DS). The binding site in the hA3G model was located by site finder module implemented in the MOE. The ligand binding region was defined as a sphere of 12 Å radius around the binding site. Molecular interactions were observed using LigX implemented in MOE. The NCI database was used for virtual screening against the predicted site of hA3G model. The database was first filtrated according to the drug-like properties of the compound using “Prepare Ligands” module in DS. The filtrated database was then screened against the predicted site of hA3G model using docking module Libdock in DS. The candidates with the values of libscore more than 90 were further evaluated in GOLD software in two steps (Gold efficiency 30% and Gold efficiency 100%). The final candidates with goldscore more than 80 were then visually inspected to check the binding mode. At the end, 26 compounds were selected and obtained from NCI.

### Plasmids, compounds and proteins

The expression vectors for Vif, hemagglutinin (HA) tagged hA3G and hA3F were previously described^2,48–50^. The compounds tested in this work were obtained from NCI. Purified hA3G (>95% purity, catalog no. 10067) were obtained through the AIDS Research and Reference Reagent Program, Division of AIDS, NIAID, National Institutes of Health.

### Cells and viruses

293 T and TZM-bl cells were cultured in DMEM (GBICO) supplemented with 10% fetal bovine serum (FBS) (GBICO). SupT1 cells were maintained in RPMI-1640 (GBICO) containing 10% FBS. H9 cells were maintained in RPMI-1640 (GBICO) containing 10% FBS. Transfections of 293 T cells were performed using Lipofectamine 2000 (Invitrogen) according to the manual from the manufacturer. To collect viral particles, the supernatants of virus-producing cells were pelleted through a 20% sucrose cushion at 35000 rpm for 60 min (Beckman). The harvested viral samples were analyzed by western blotting.

### Western blots

Western blots were probed with monoclonal antibodies against HIV-1 p24 (NIH), HA (Santa Cruz), β-actin (Abcam) and hA3G (Abcam). Detection of proteins was performed by enhanced chemiluminescence (Millipore), using secondary antibodies anti-mouse (for β-actin) and anti-rabbit (for P24 and HA), both were purchased from Santa Cruz Biotechnology Inc. Bands in western blots were quantitated using ChemiDoc TMMP (Bio-Rad) automated digitizing system and Image J software.

### HIV-1 Infectivity assay

To assess the effect of the four small compounds on HIV-1 infectivity, the experiments were carried out in H9 and SupT1 cells. H9 and SupT1 cells were infected with wild-type HIV-1(NL4-3), respectively, in the presence of the compounds at various concentrations. The infectivity of nascent viruses in the supernatant was measured by infecting the TZM-bl indicator cell for 48 hours (hr). To assess the effect on the pseudotyped HIV-1, 293 T cells were co-transfected with 300 ng pNL4-3Luc(R-E-), 200 ng VSVG and 200 ng hA3G-HA (or pcDNA3.1) plasmid DNA in 6-wellplates. After 12 hr, the media was changed and IMB-301 was added. Thirty-six hours later, the supernatants were collected and filtered through a 0.45 µm filter, and then used to infect the SupT1 cells (1 × 10^5^) in 96-well plates. Forty-eight hours later, SupT1 cells were lysed and firefly luciferase activities were determined using a firefly Luciferase Assay System (Promega). Equal volumes of DMSO were added into the culture medium, which contain different concentrations of the compounds, in order to keep constant final DMSO concentration as 1% (v/v).

### Cytotoxicity assay

The cytotoxicity of IMB-301 was measured using the CCK8 Assay Kit (Beyotime). The kit provides an assay that distinguishes metabolically active cells from injured cells and dead cells. SupT1 or H9 cells were treated with IMB-301 at various concentrations. DMSO treated cells were used as the control. Twenty-four hours post treatment, the samples were subjected to Live/Dead Cell Vitality Assay Kit following the manufacturer manual. The samples were analyzed at OD 450. Equal volumes of DMSO were added into the culture medium, which contain different concentrations of the compounds, in order to keep constant final DMSO concentration as 1% (v/v).

### Co-immunoprecipitation

For hA3G and Vif IP assay, 293 T cells were seeded in 10 cm dishes. Twenty-four hours later, the cells were co-transfected with 1 µg Vif and 3 µg hA3G-HA. Another 24 hr later, the cells were treated with DMSO or 10 µM IMB-301 for 24 hr. Lastly, the cells were collected and lysated in NP-40-containing buffer for 30 min on ice. Cell lysates were centrifugated at 10,000x g for 10 min at 4 °C and supernatant was transferred to a fresh 1.5 ml tube on ice. The cell lysates were incubated with anti-HA rabbit antibody for 3 hr at 4 °C. Thirty microliters of proteinA-Agarose (Santa Cruz) was added and incubated overnight at 4 °C. The beads were washed 4 times with lysis buffer (cold) and at last boiled in 40 µl sample buffer for 5 to 10 min. The samples were analyzed by Western blotting.

### DNA hypermutation assay

293 T cells were co-transfected with 300 ng pNL4-3(R-E-) (or pNL4-3-vif-), 200 ng VSVG and 300 ng hA3G-HA in6-well-plate. After 12 hr, the media was changed and IMB-301 was added. Thirty-six hours later, culture supernatants containing released virus were used to infect TZM-bl cell for 4 hr.The infected TZM-bl cells were washed twice with PBS then incubated at 37 °C, 5% CO2 for 16 hr. DNA was isolated using DNeasy DNA isolation kit (Qiagen). About 700 bp DNA fragment of HIV-1 was amplified with Taq DNA polymerase (Invitrogen) using the primers HIV-1-F, 5′-AGGCAGCTGTAG ATATTAGCCAC, and HIV-1-R, 5′-GTATGAGGGATCTCTAGCTACCA^35^. The PCR products were cloned into the TA-cloning vector (Invitrogen). The nucleotide sequences of individual clones from each infected culture sample were determined. Statistical significance was determined using the GraphPad Prism software.

### Real-time PCR

#### Group one (late stages)

293 T cells were co-transfected with 300 ng pNL4-3(R-E-), 200 ng VSVG and 300 ng hA3G-HA in a 6-well-plate. After 12 hr, the media was changed and compounds (MG132, EFV, RAL, IMB-293, IMB-301, IMB-350, IMB-945, and IMB-26) were added. Thirty-six hours later, culture supernatants containing the released viruses were used to infect293T cells. Forty-eight hours later, total DNA was isolated using the DNeasy DNA isolation kit (Qiagen).Group two: 293T cells were transfected with 300 ng hA3G-HA in 6-well-plate and then were infected by VSV-G pseudotyped HIV-1, followed by the treatment with the above compounds. Forty-eight hours post infection, total DNA was isolated using the DNeasy DNA isolation kit (Qiagen). U5-gag, Alu-LTR and IN represent plus strand DNA, transfer DNA and integrated proviral cDNA. Four pairs of primers were used: U5-gag-forward: TGTGTGCCCGTCTGTTGTGTGA; U5-gag-reverse: TCAGCAAGCCGAGTCCTGCGT; Alu-LTR-forward: TCCCAGCTACTCGGGAGGCTGAGG; Alu-LTR-reverse: AGG CAAGCTTTATTGAGGCTTAAGC; IN-forward: CACACACAAGGCTACTTCCCT; IN-reverse: TAGCCACTCCCCAGTCCCGCCC; GAPDH-forward: GAAGGTGAAGGTCGGAGT; GAPDH-reverse: GAAGATGGTGATGGGATTTC^35,51^. QPCR results were normalized using the GAPDH DNA levels and calculated by the 2^−△△Ct^ comparative method.

### Octet binding

The Octet RED (ForteBio, Inc., CA, USA), equipped with super streptavidin biosensor chips (ForteBio), was used for the analysis of small molecule protein interactions in fluidics free system^52^. The binding of IMB-301 to the purified hA3G protein was performed by ForteBio Inc. Company (www.fortebio.com). The purified hA3G was biotinylated(EZ-Link® NHS-Biotin Reagents, Cat.# 21343, Thermo) by 3:1, and then incubated for 1 hr at room temperature. Superstreptavidin biosensors (Forte´Bio Inc., Menlo Park, CA) were coated in a solution containing 1 μM of biotinylated protein for 1 hr at room temperature. A duplicate set of sensors was incubated in an assay buffer with 5% dimethyl sulfoxide without protein for use as a background binding control. Both sets of sensors were blocked with a solution of 10 mg/ml biocytin for 5 min at room temperature. A negative control of 5% dimethyl sulfoxide was also used. Binding of samples to coated and uncoated reference sensors was measured over 120 s. Data analysis on the Forte’Bio Octet RED instrument was performed using a double reference subtraction (sample and sensor references) in the Forte’Bio data analysis software.

## Electronic supplementary material

Supplementary Electrophoretic gels and blots (original)

Supplemental Material
