# Supplementary material for: Identification of small molecule compounds targeting the interaction of HIV-1 Vif and human APOBEC3G by virtual screening and biological evaluation
Source: Sci Rep. 2018 May 23;8:8067. doi: 10.1038/s41598-018-26318-3 (PMC5966509; doi:10.1038/s41598-018-26318-3)
Supplement: Supplementary file 1 — Supplementary Electrophoretic gels and blots (original) [file 41598_2018_26318_MOESM1_ESM.docx]

**Identification of small molecule compounds targeting the interaction of HIV-1 Vif and human APOBEC3G by virtual screening and biological evaluation**

Ling Ma^1^, Zhixin Zhang^1^, Zhenlong Liu^2^, Qinghua Pan^2^, Jing Wang^1^, Xiaoyu Li^1*^, Fei Guo^3^, Chen Liang^2^, Laixing Hu^1^，Jinming Zhou^1^*，Shan Cen^1^*

^1^Institute of Medicinal Biotechnology, Chinese Academy of Medical Sciences & Peking Union Medical College, Beijing, China.

^2^Lady Davis Institute for Medical Research, Jewish General Hospital, McGill University, Montreal, QC, Canada.

^3^Institute of Pathogen Biology, Chinese Academy of Medical Sciences & Peking Union Medical College, Beijing, China.

Correspondence and requests for materials should be addressed to X.Y.L. (email: xiaoyuli@imb.pumc.edu.cn) or J.M.Z. (email: zhou_jim@imb.pumc.edu.cn) or S.C. (email: shancen@imb.pumc.edu.cn)

**Electrophoretic gels and blots (original) for SREP-17-31557**

**12% of gel**

**Figure1**

**Fig1B**


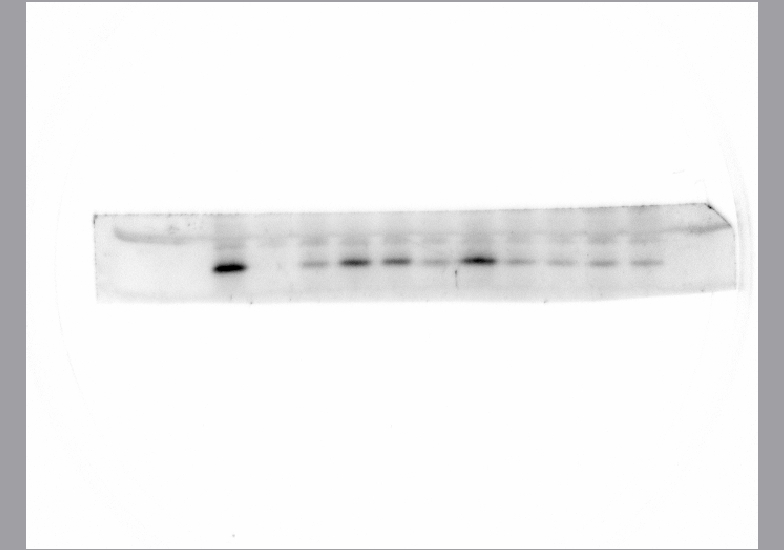


DMSO

DMSO

DMSO

IMB-293 IMB-301

IMB-342

IMB-350

IMB-351

IMB-463

IMB-363

IMB404

**hA3G-HA-1**

42KD


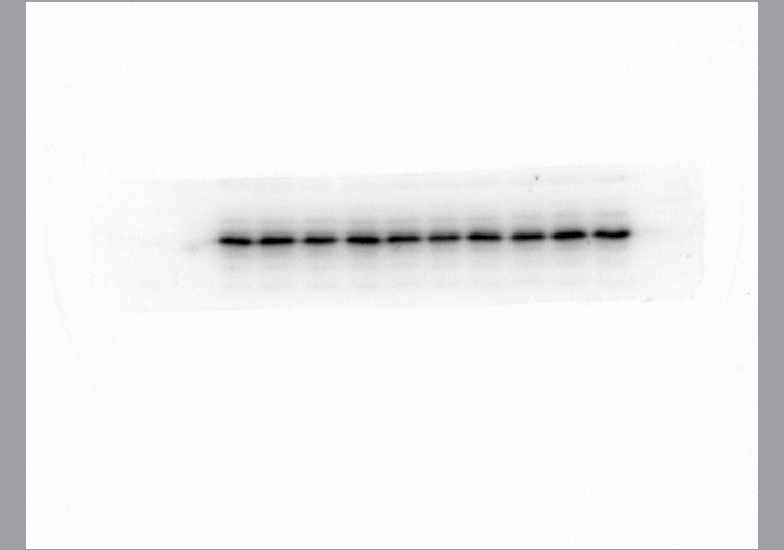


**Vif-1**

23KD

DMSO

DMSO

DMSO

IMB-293 IMB-301

IMB-342

IMB-350

IMB-351

IMB-463

IMB-363

IMB404


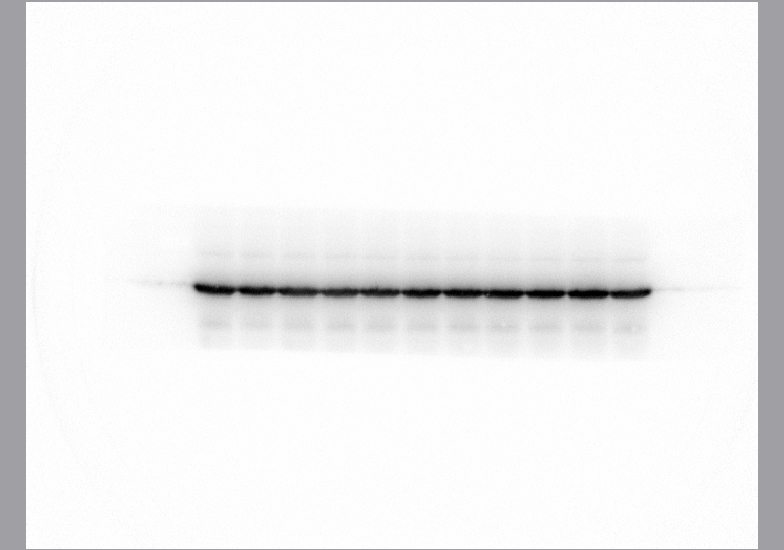


43KD

**actin-1**

DMSO

DMSO

DMSO

IMB-293 IMB-301

IMB-342

IMB-350

IMB-351

IMB-463

IMB-363

IMB404


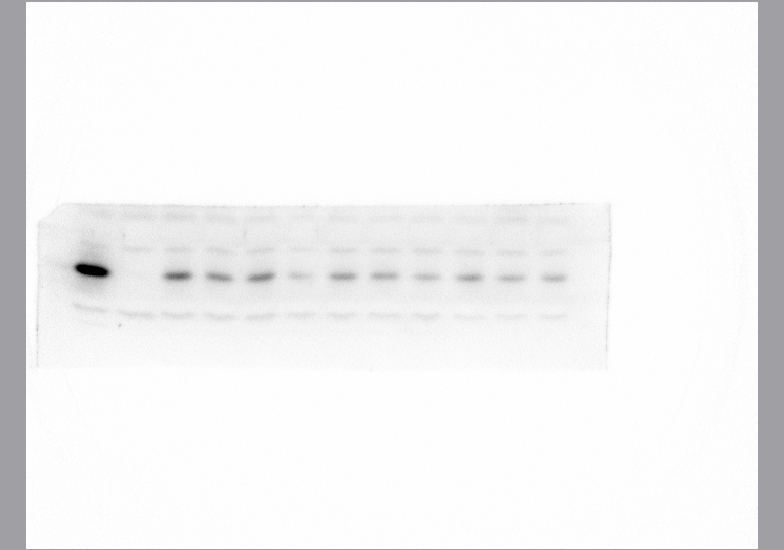


DMSO

DMSO

DMSO

IMB-146

IMB-169

IMB-166

IMB-174

IMB-211

IMB-212

IMB-247

IMB-279

IMB-281

42KD

**hA3G-HA-2**


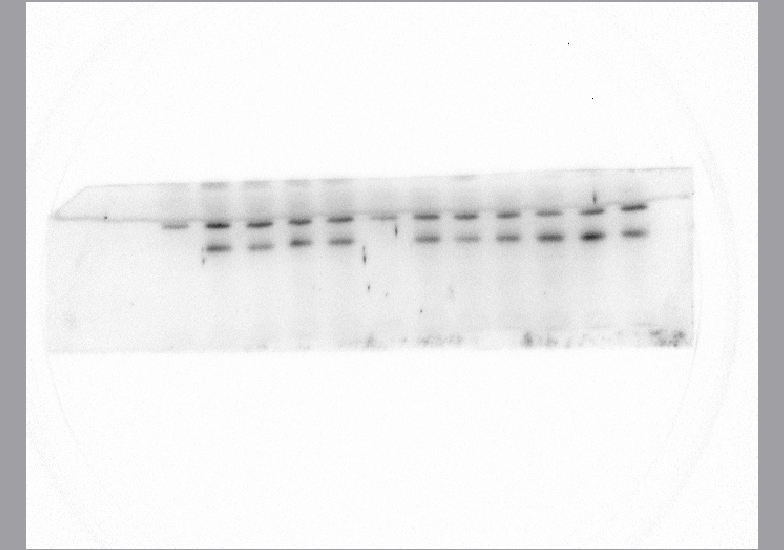


DMSO

DMSO

DMSO

IMB-146

IMB-169

IMB-166

IMB-174

IMB-211

IMB-212

IMB-247

IMB-279

IMB-281

**Vif-2**

23KD


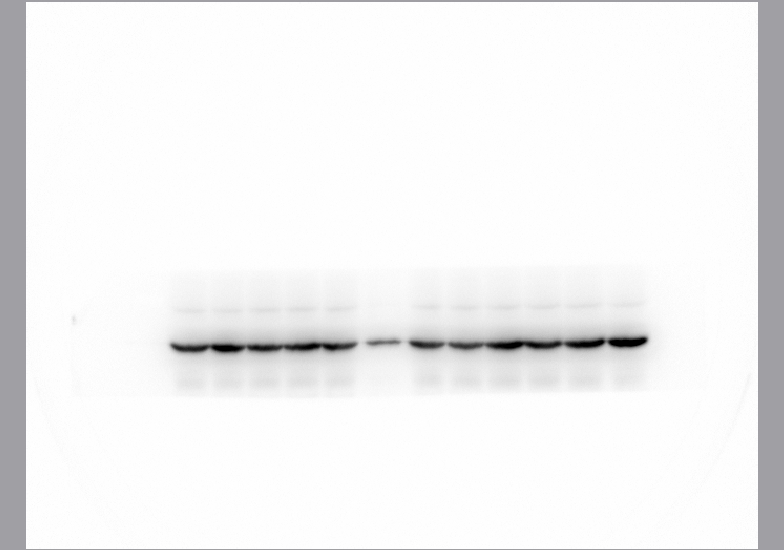


DMSO

DMSO

DMSO

IMB-146

IMB-169

IMB-166

IMB-174

IMB-211

IMB-212

IMB-247

IMB-279

IMB-281

43KD

**actin-2**


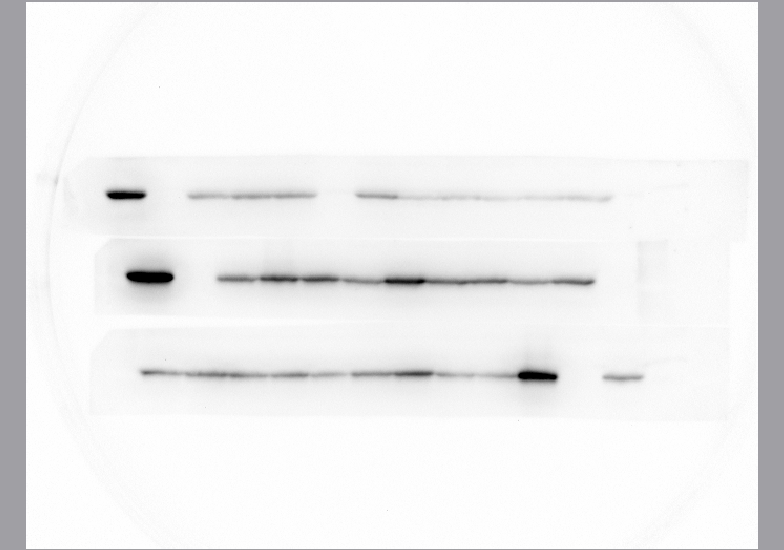


42KD

**hA3G-HA-3 (Reverse order)**

IMB-334 IMB-365

IMB-479

IMB-509

IMB-810

IMB-916

IMB-945

IMB-131

IMB-137

DMSO

DMSO

DMSO


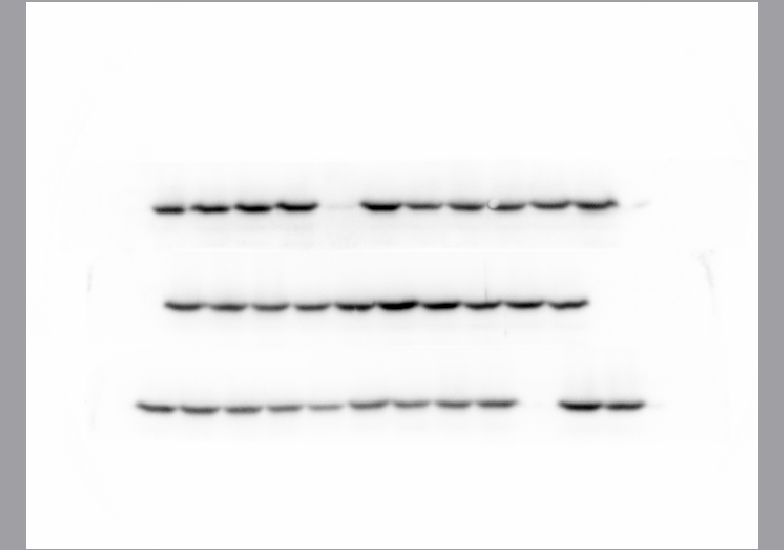

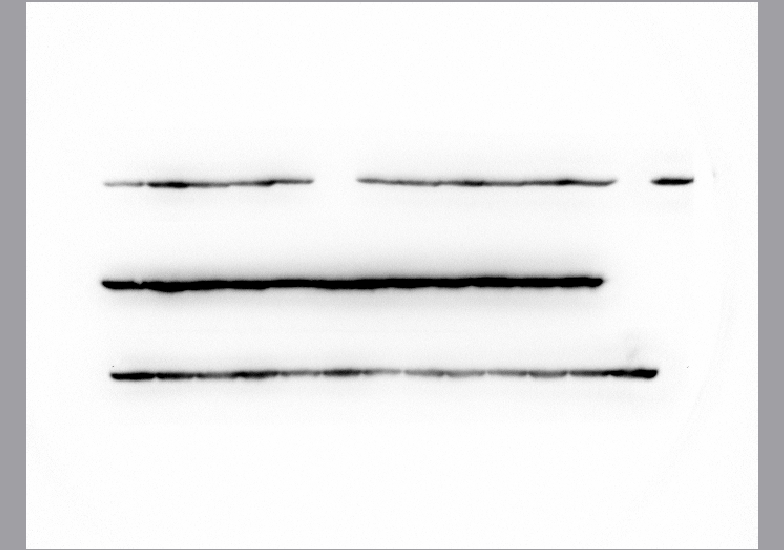


23KD

**Vif-3 (Reverse order)**

IMB-334 IMB-365

IMB-479

IMB-509

IMB-810

IMB-916

IMB-945

IMB-131

IMB-137

DMSO

DMSO

DMSO

**Actin-3 (Reverse order)**

IMB-334 IMB-365

IMB-479

IMB-509

IMB-810

IMB-916

IMB-945

IMB-131

IMB-137

DMSO

DMSO

DMSO

DMSO

DMSO

DMSO

IMB-293 IMB-301

IMB-342

IMB-350

IMB-351

IMB-463

IMB-363

IMB404

43KD

43KD

**Actin-1**

**Figure2**

**Fig2A**


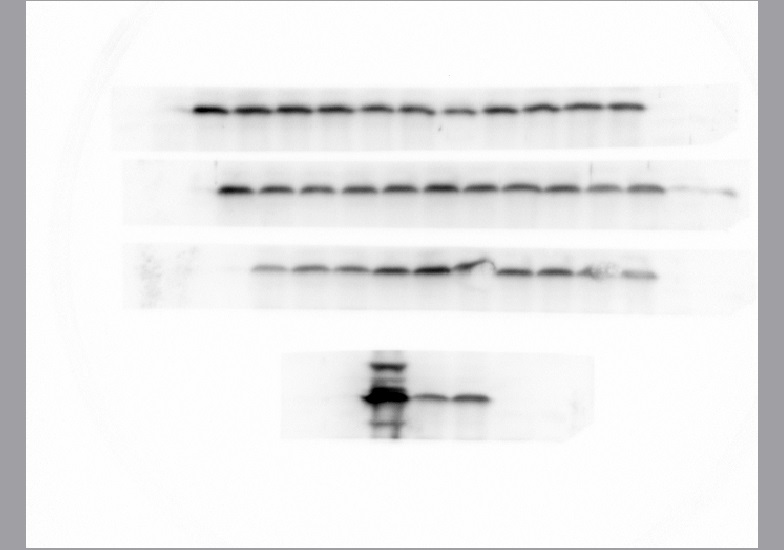

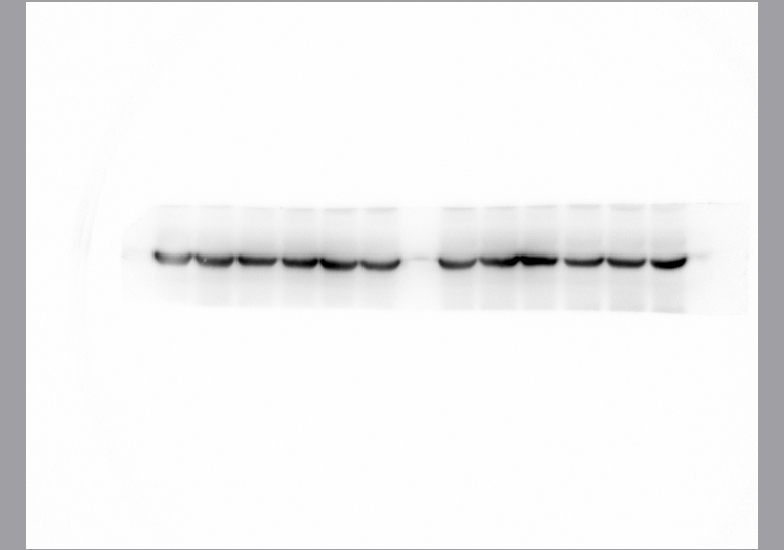


**hA3G-HA**

DMSO

IMB-293

IMB-301

IMB-350

IMB-945

MOCK

DMSO

IMB-293

IMB-301

IMB-350

IMB-945

MOCK

43KD

42KD

**Actin**

**Fig 2B**

**
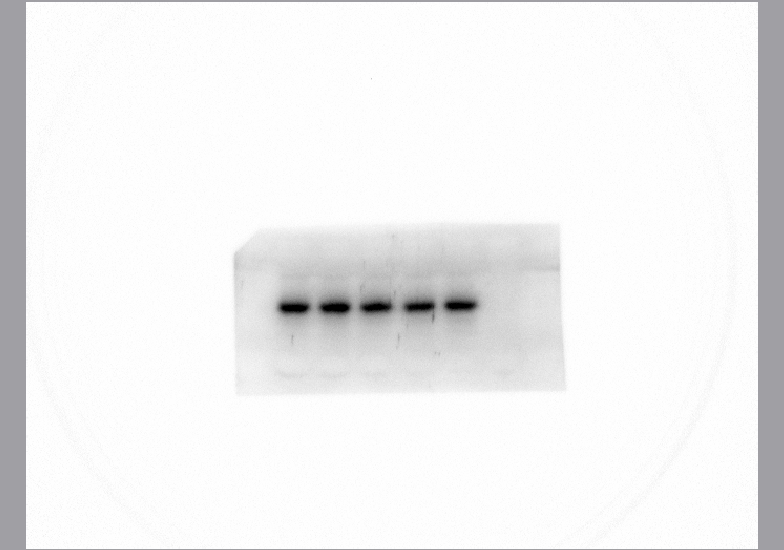
**

DMSO

IMB-293

IMB-301

IMB-350

IMB-945

MOCK

23KD

**Vif**

**
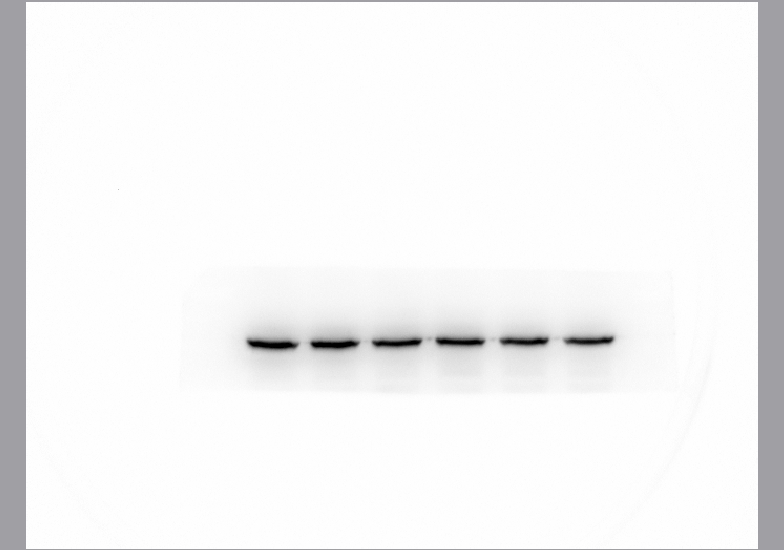
**

DMSO

IMB-293

IMB-301

IMB-350

IMB-945

MOCK

43KD

**Actin**

**Fig2C**

**
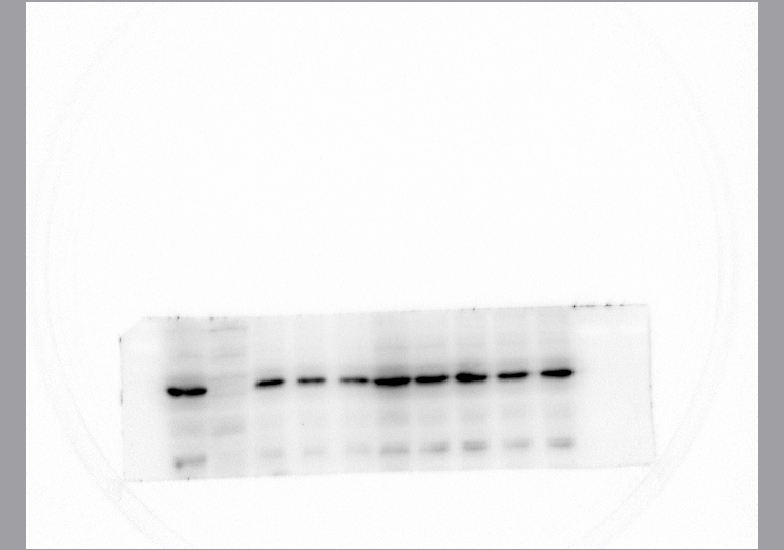
**

**hA3G-HA-input**

DMSO

DMSO

IgG

PBS

DMSO

MG132

IMB-293

IMB-301

IMB-350

IMB-945

42KD

**
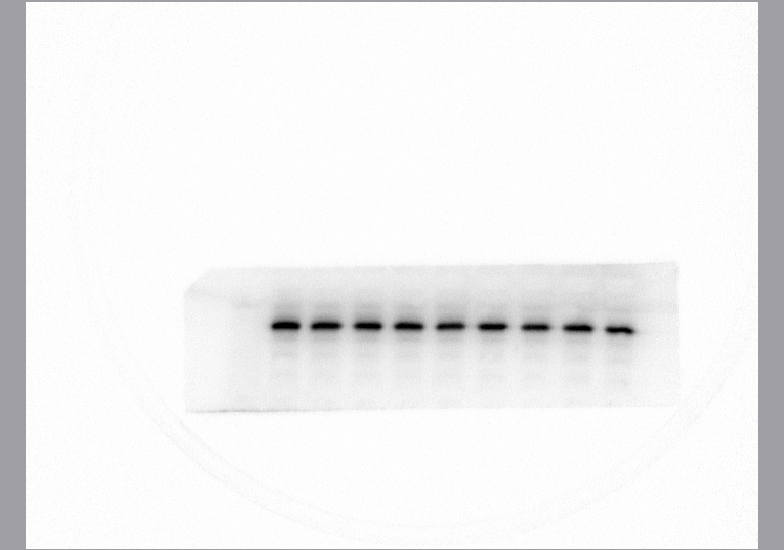
**

DMSO

DMSO

IgG

PBS

DMSO

MG132

IMB-293

IMB-301

IMB-350

IMB-945

23KD

**Vif-input**

**
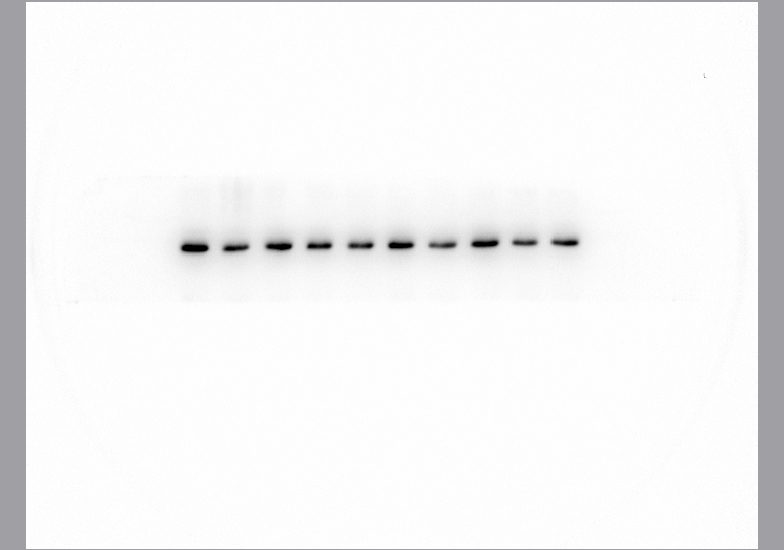
**

DMSO

DMSO

IgG

PBS

DMSO

MG132

IMB-293

IMB-301

IMB-350

IMB-945

**Actin-input**

43KD


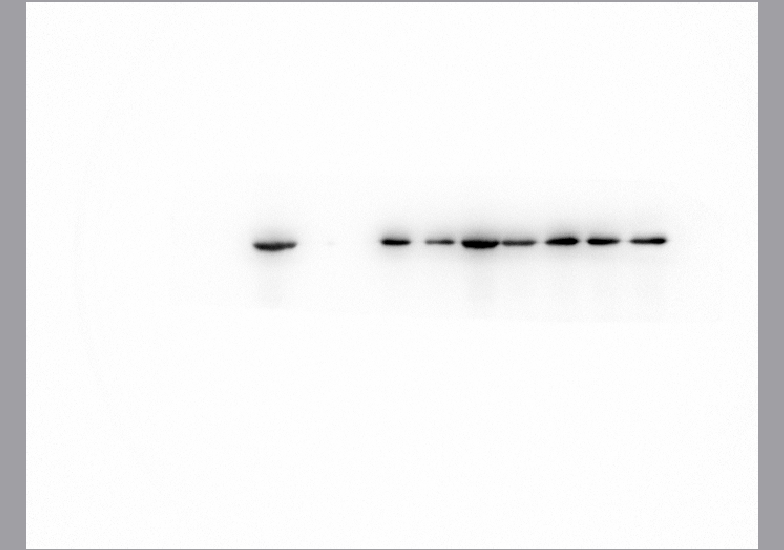


**hA3G-HA-ip**

DMSO

DMSO

IgG

PBS

DMSO

MG132

IMB-293

IMB-301

IMB-350

IMB-945

42KD


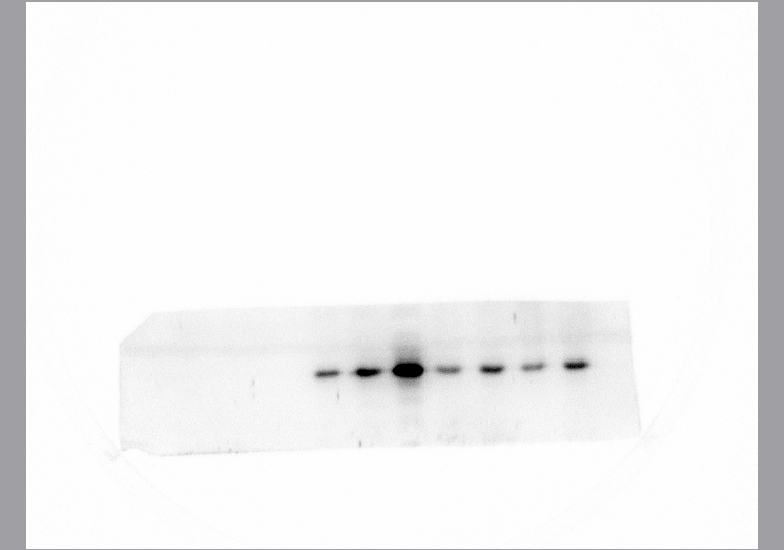


DMSO

DMSO

IgG

PBS

DMSO

MG132

IMB-293

IMB-301

IMB-350

IMB-945

**Vif-ip**

23KD

**Fig 4**

**Fig4A**


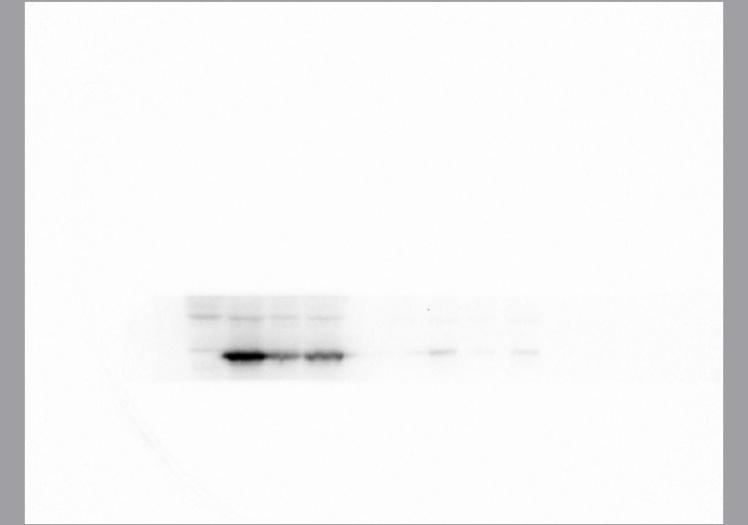

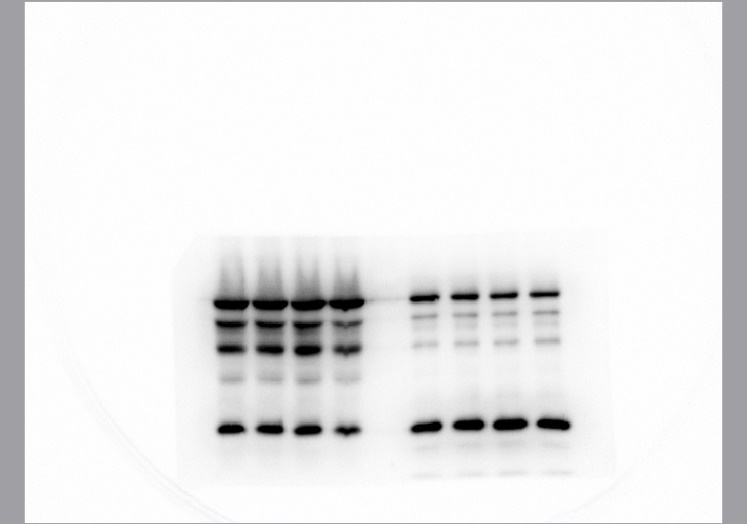

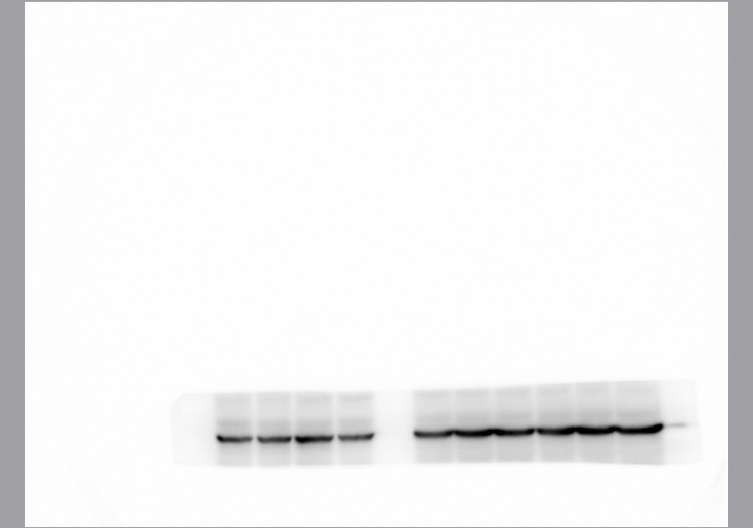

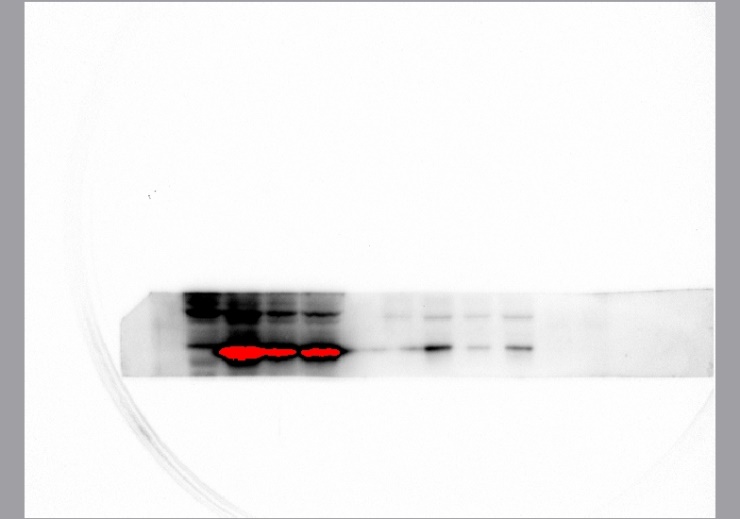


**hA3G-HA-cell**

DMSO

DMSO

DMSO

IMB-301

DMSO

DMSO

DMSO

IMB-301

DMSO

DMSO

DMSO

IMB-301

43KD

24KD

55KD

42KD

**Actin-cell**

**P24-cell**


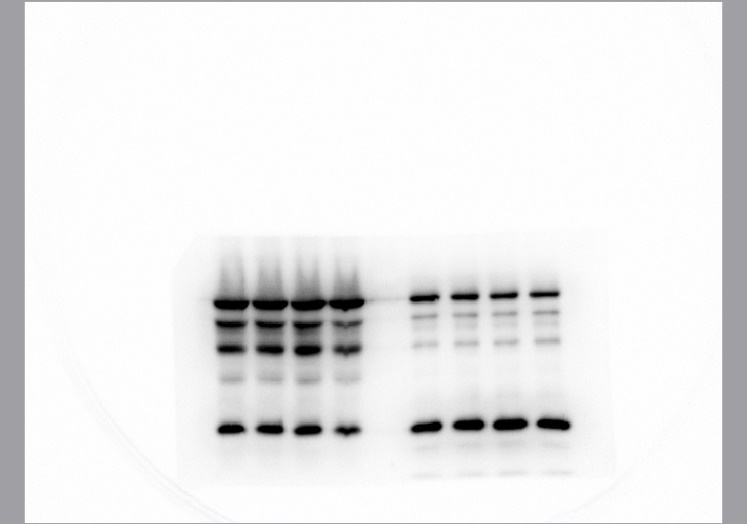


**hA3G-HA-virus**

DMSO

DMSO

DMSO

IMB-301

DMSO

DMSO

DMSO

IMB-301

24KD

55KD

42KD

**P24-virus**

**Figture5**

**Fig5A**

**
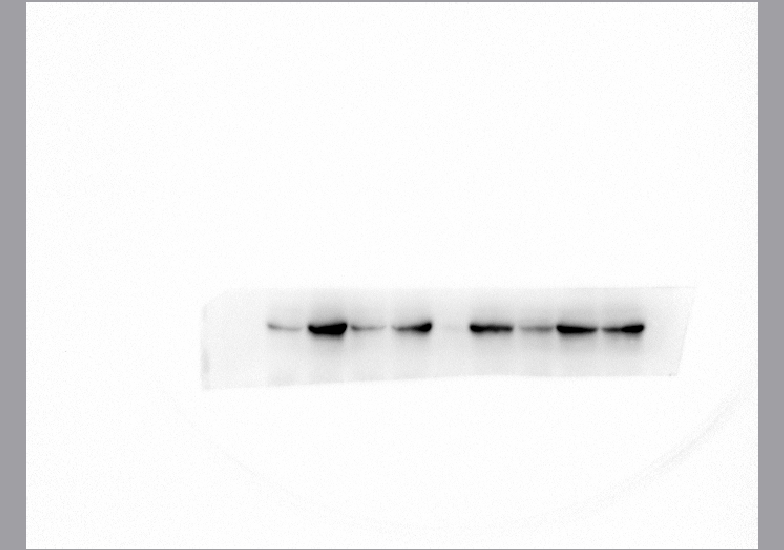

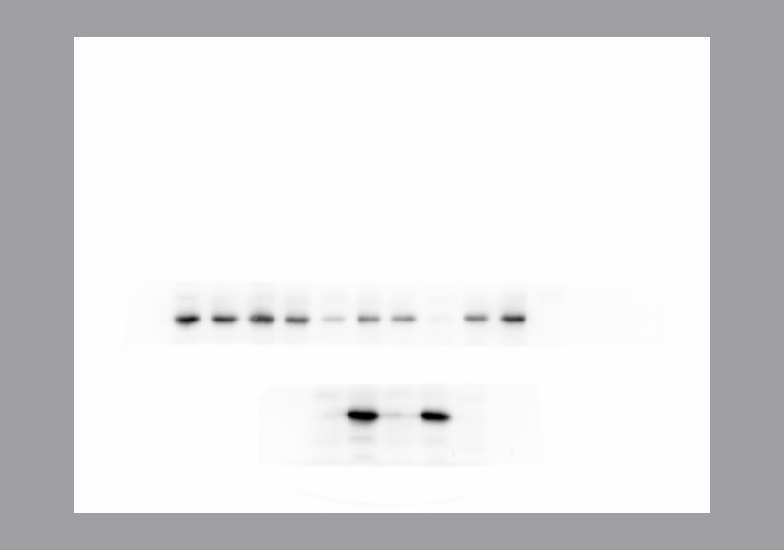

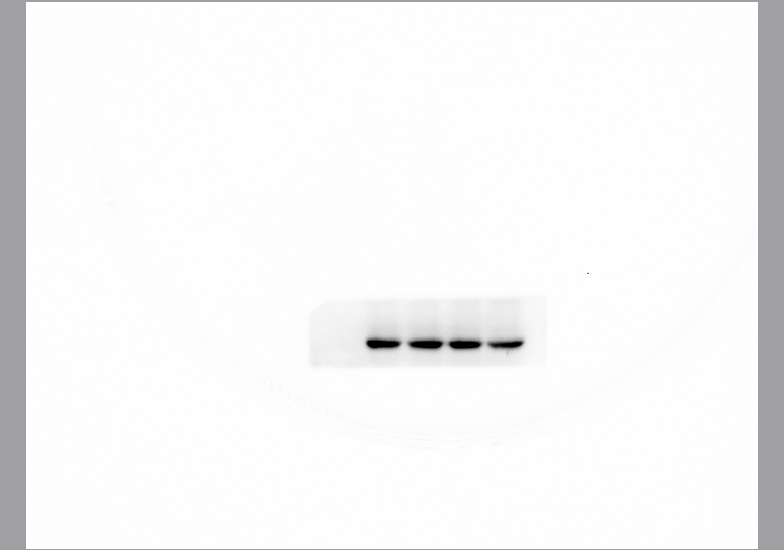
**

**hA3G-HA**

DMSO

DMSO

IMB-301

IMB-301

DMSO

DMSO

IMB-301

IMB-301

DMSO

DMSO

IMB-301

IMB-301

43KD

23KD

42KD

**Actin**

**Vif**

**Fig5B**

**
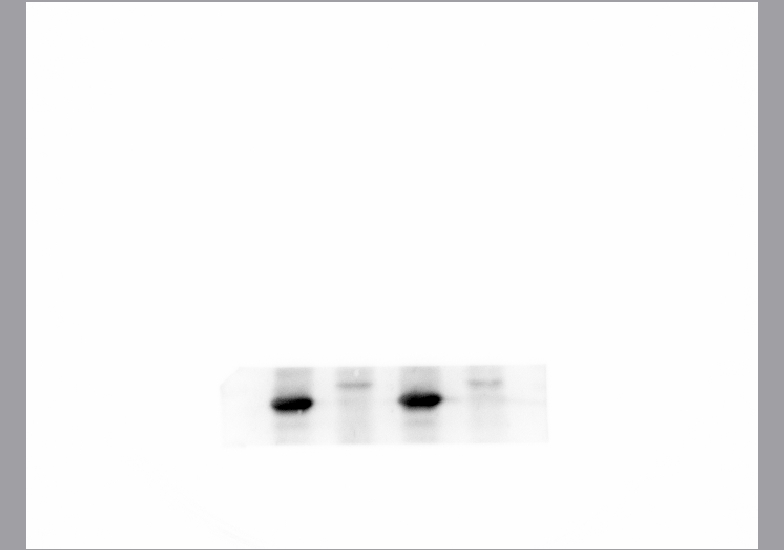

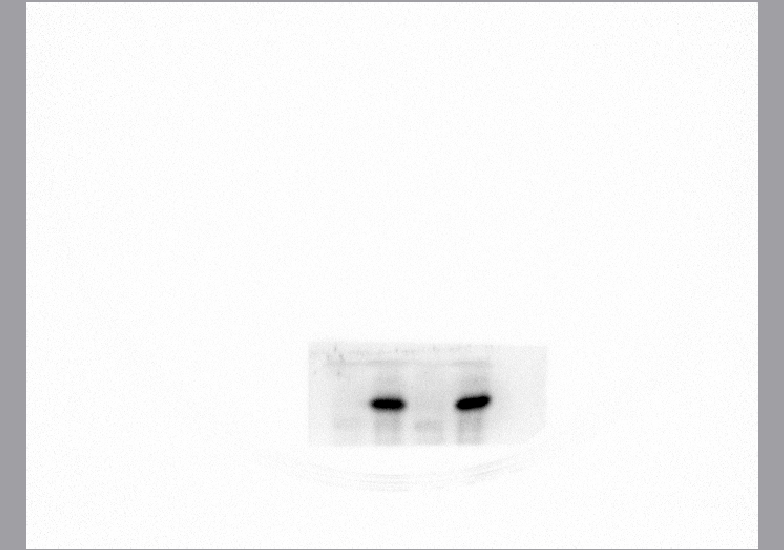

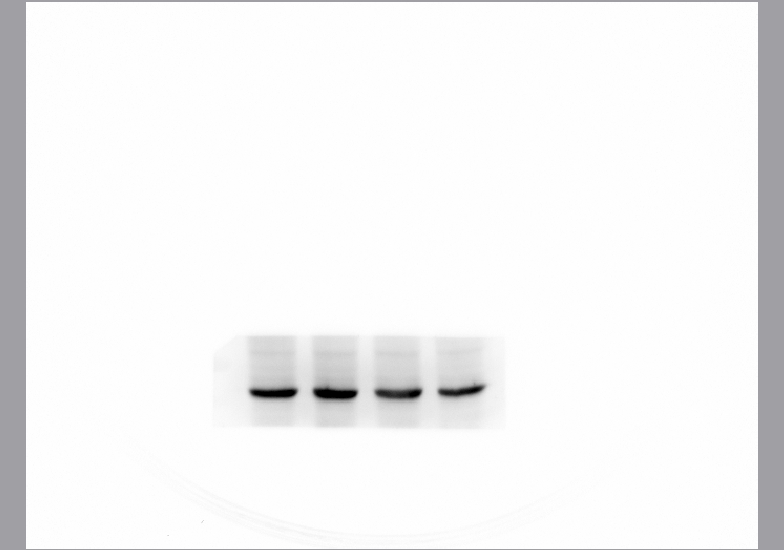
**

**hA3F-HA**

DMSO

DMSO

IMB-301

IMB-301

DMSO

DMSO

IMB-301

IMB-301

DMSO

DMSO

IMB-301

IMB-301

43KD

40KD

**Vif**

**Actin**

23KD

**Figure S1 (B)**


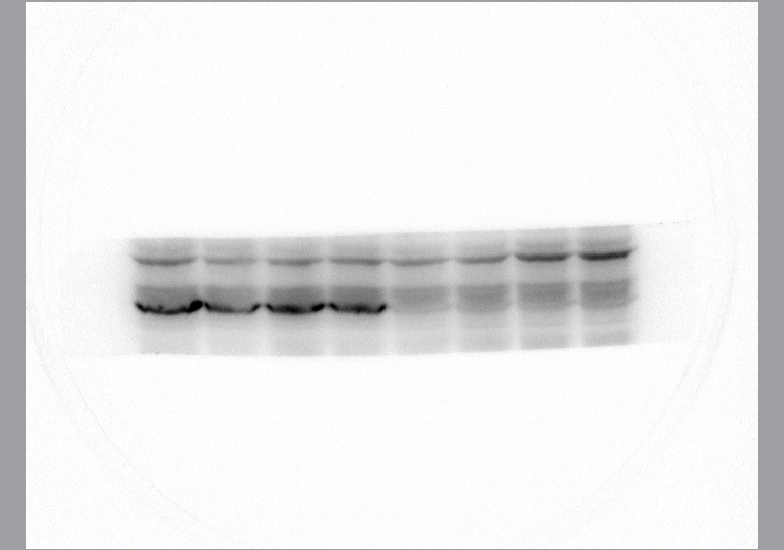


DMSO

IMB301-10Μm

IMB301-20μM

IMB301-40μM

DMSO

IMB301-10μM

IMB301-20μM

IMB301-40μM

**hA3G**

43KD


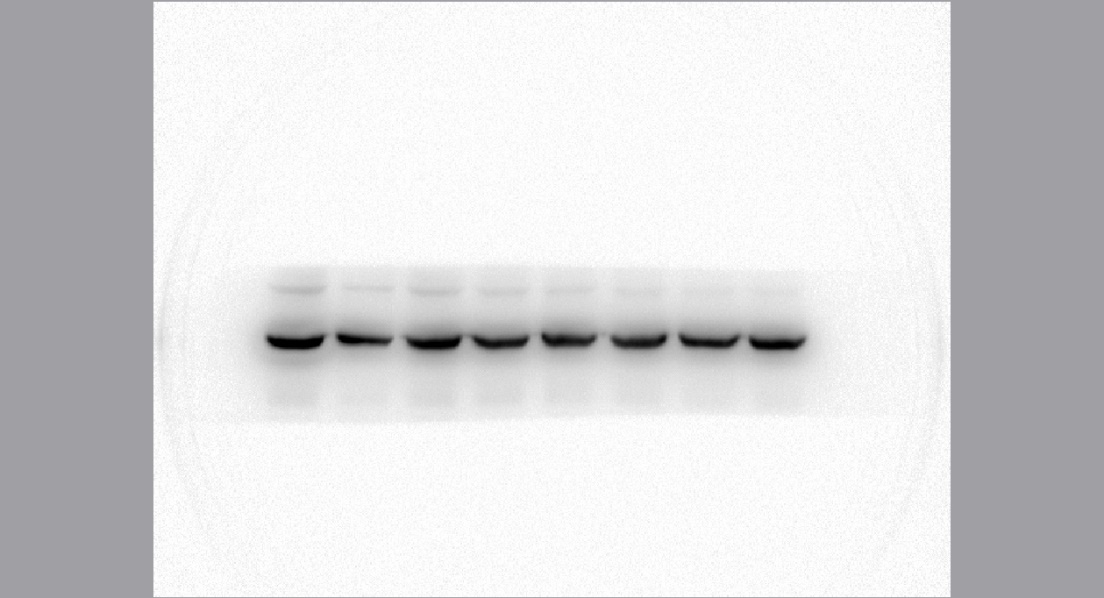


DMSO

IMB301-10Μm

IMB301-20μM

IMB301-40μM

DMSO

IMB301-10μM

IMB301-20μM

IMB301-40μM

**Actin**

43KD

**Figure S6**


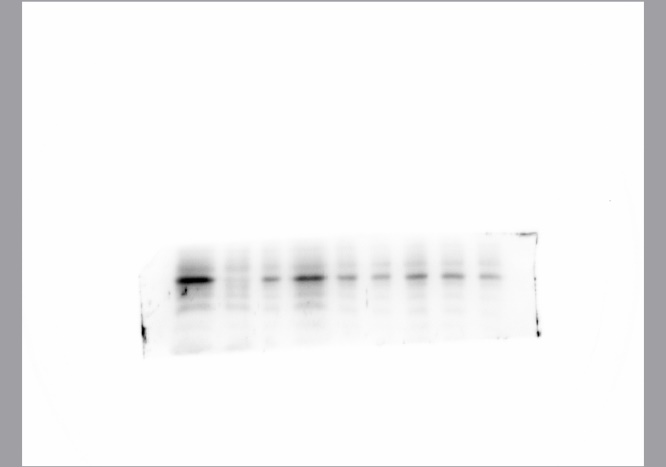

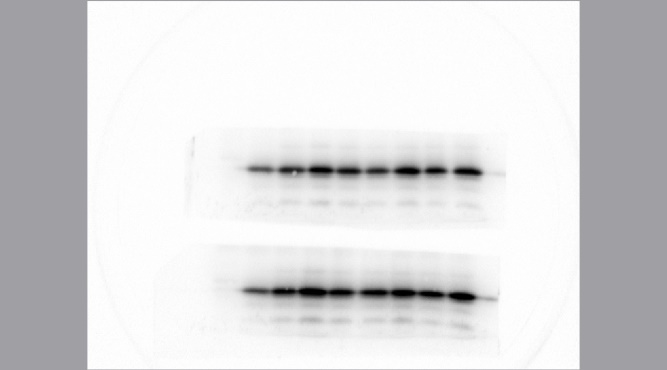

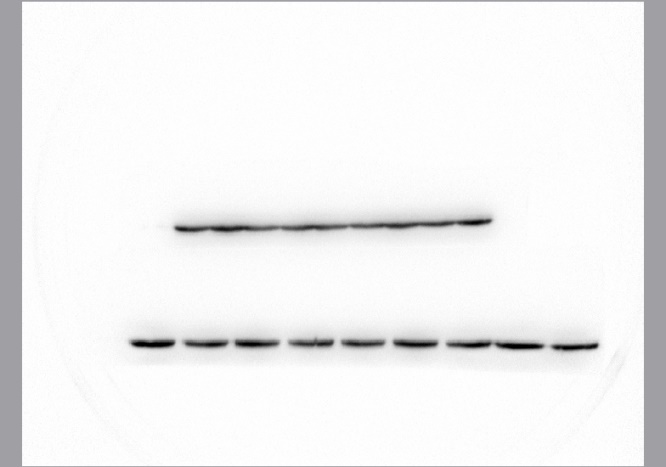


**hA3F-HA**

DMSO

DMSO

DMSO

MG132

IMB-293

IMB-301

IMB-350

IMB-945

IMB-26

DMSO

DMSO

DMSO

MG132

IMB-293

IMB-301

IMB-350

IMB-945

IMB-26

DMSO

DMSO

DMSO

MG132

IMB-293

IMB-301

IMB-350

IMB-945

IMB-26

43KD

23KD

40KD

**Actin**

**Vif**
