# Supplementary material for: Identification of small molecule compounds targeting the interaction of HIV-1 Vif and human APOBEC3G by virtual screening and biological evaluation
Source: Sci Rep. 2018 May 23;8:8067. doi: 10.1038/s41598-018-26318-3 (PMC5966509; doi:10.1038/s41598-018-26318-3)
Supplement: Supplementary file 2 — Supplemental Material [file 41598_2018_26318_MOESM2_ESM.pdf]

Identification of small molecule compounds targeting the interaction of HIV-1 Vif and human APOBEC3G by virtual screening and biological evaluation

Ling Ma[1], Zhixin Zhang[1], Zhenlong Liu[2], Qinghua Pan[2], Jing Wang[1], Xiaoyu Li[1*], Fei Guo[3], Chen Liang[2], Laixing Hu[1], Jinming Zhou[1*], Shan Cen[1*]

[1]Institute of Medicinal Biotechnology, Chinese Academy of Medical Sciences & Peking Union Medical College, Beijing, China.

[2]Lady Davis Institute for Medical Research, Jewish General Hospital, McGill University, Montreal, QC, Canada.

[3]Institute of Pathogen Biology, Chinese Academy of Medical Sciences & Peking Union Medical College, Beijing, China.

Correspondence and requests for materials should be addressed to X.Y.L. (email: xiaoyuli@imb.pumc.edu.cn) or J.M.Z. (email: zhou_jim@imb.pumc.edu.cn) or S.C. (email: shancen@imb.pumc.edu.cn)

Table of content

Figure S1. Effect of IMB-301 on expression of endogenous hA3G in both H9 and SupT1 cells

Figure S2. The hypermuation rate in viral genome gradually was increased by IMB-301in dose-dependent manner

Figure S3. Octet binding analysis of IMB-293, IMB-350, IMB-945 and hA3G

Figure S4. The superimposion of the homology structure of A3G-NTDand NMR structure of A3G-NTD.

Figure S5. The predicted binding sites in the homology structure of A3G-NTD and NMR structure of A3G-NTD.

Figure S6. IMB-293, IMB-301, IMB-350, and IMB-945 specifically rescue hA3G degradation rather than hA3Fin presence of Vif

Table S1. The similarity analysis between IMB-301 and other active compounds. The similarity was calculated using Open Babel.

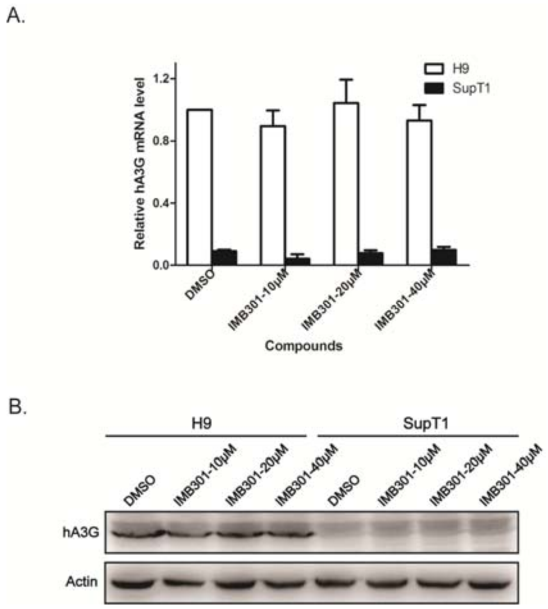

Figure S1. H9 and SupT1 cells were seeded in 6-well-plate, and treated with IMB301 at different concentrations (10μM, 20μM, and 40μM). 48 hours later, cells were collected for (A) qRT-PCR and (B) western blots assay. hA3G primers for qRT-PCR:
F'-TTACCTGCTTCACCTCCTGG
R'-TCATCTAGTCCATCCCAGGG

A.

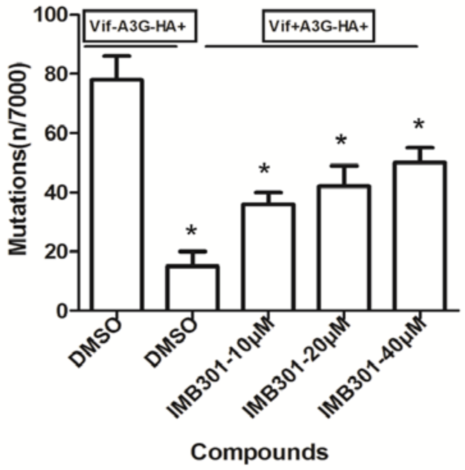

Figure S2. 293T cells were co-transfected with 300ng pNL4-3(R-E-)Vif+/-, 200ng VSVG and 200ng hA3G-HA (or pcDNA3.1) in 6-well-plate. After 12 h, the media was changed and IMB-301 was added at different concentrations (10μM, 20μM, 40μM). 36h later, the supernatants were collected and then used to infect the TZM-bl cells (1×105) in 6-well plates. 48 hours later, total DNA was isolated using DNeasy DNA isolation kit (Qiagen). About 700 bp DNA fragment of HIV-1 was amplified with Taq DNA polymerase (Invitrogen) using the primers HIV-1-F, 5'-AGGCAGCTGTAG ATATTAGCCAC, and HIV-1-R, 5'-GTATGAGGGATCTCTAGCTACCA36. The PCR products were cloned into the TA-cloning vector (Invitrogen). The nucleotide sequences of individual clones from each infected culture sample were determined. *Statistical significance was determined using the GraphPad Prism software ($P<0.05$)

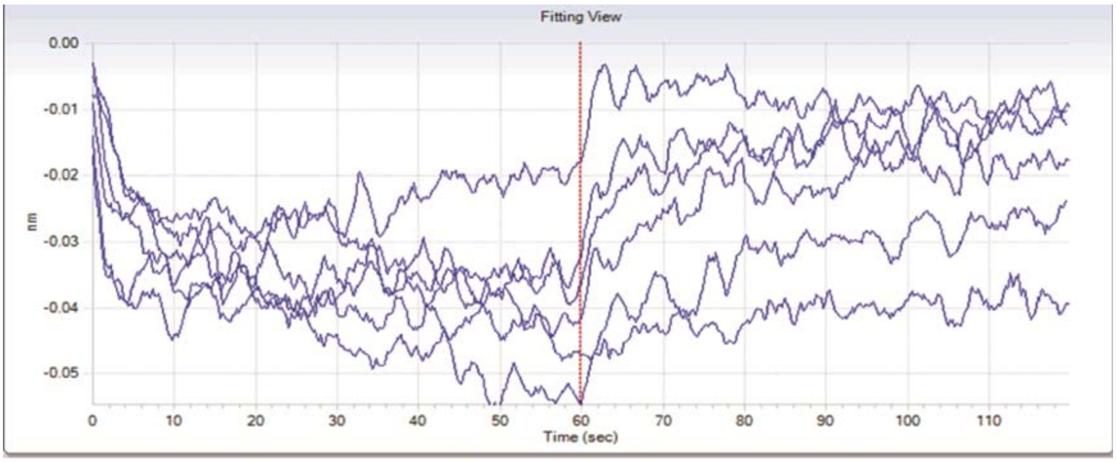

IMB-293

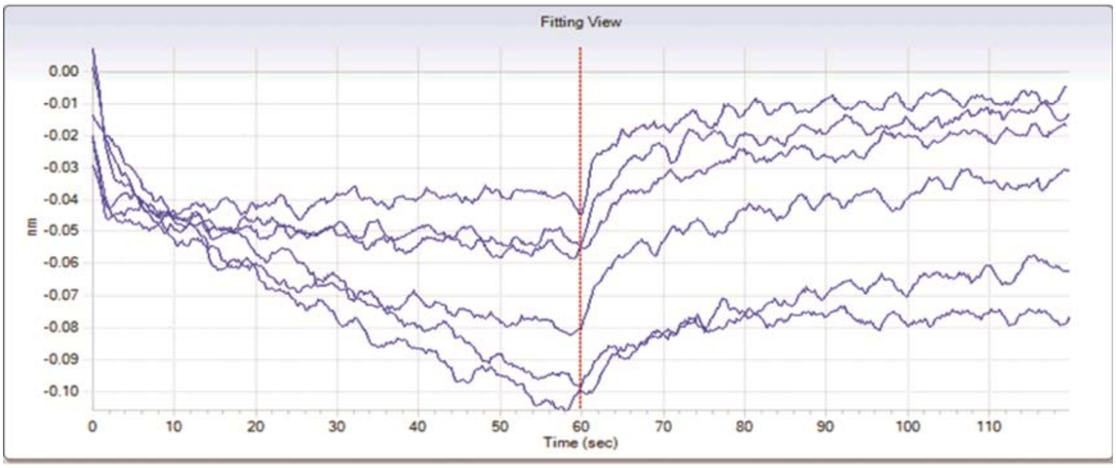

IMB-350

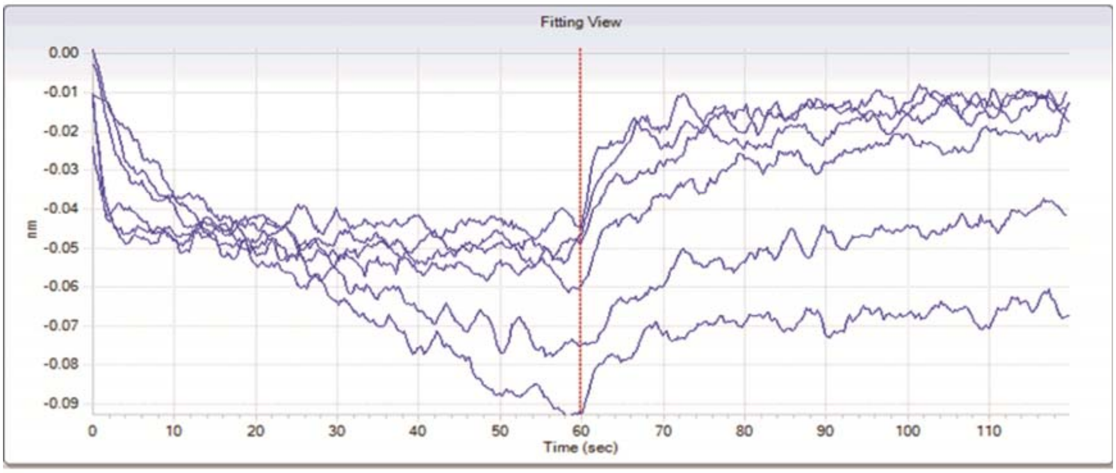

IMB-945

Figure S3. Octet binding analysis of IMB-293, IMB-350, IMB-945 and hA3G has been performed respectively. There is no binding signal shown.

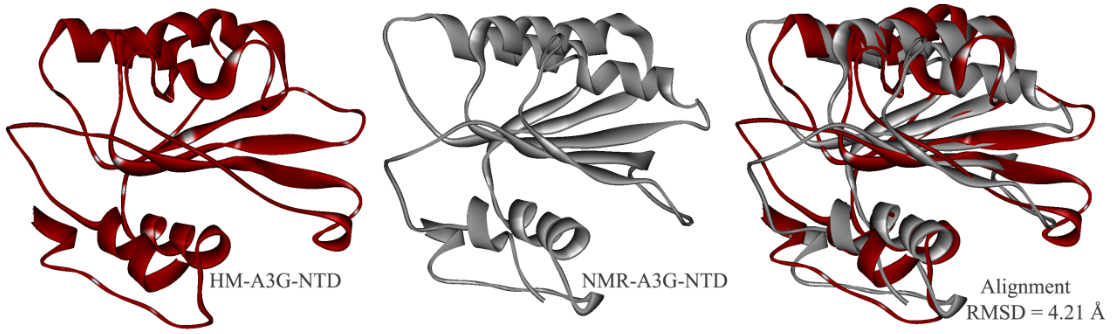

Figure S4. The superimposition of the homology structure of A3G-NTD (HM-A3G-NTD) and NMR structure of A3G-NTD (NMR-A3G-NTD) in Discovery Studio gives a RMSD value of 4.21 Å for overall structure.

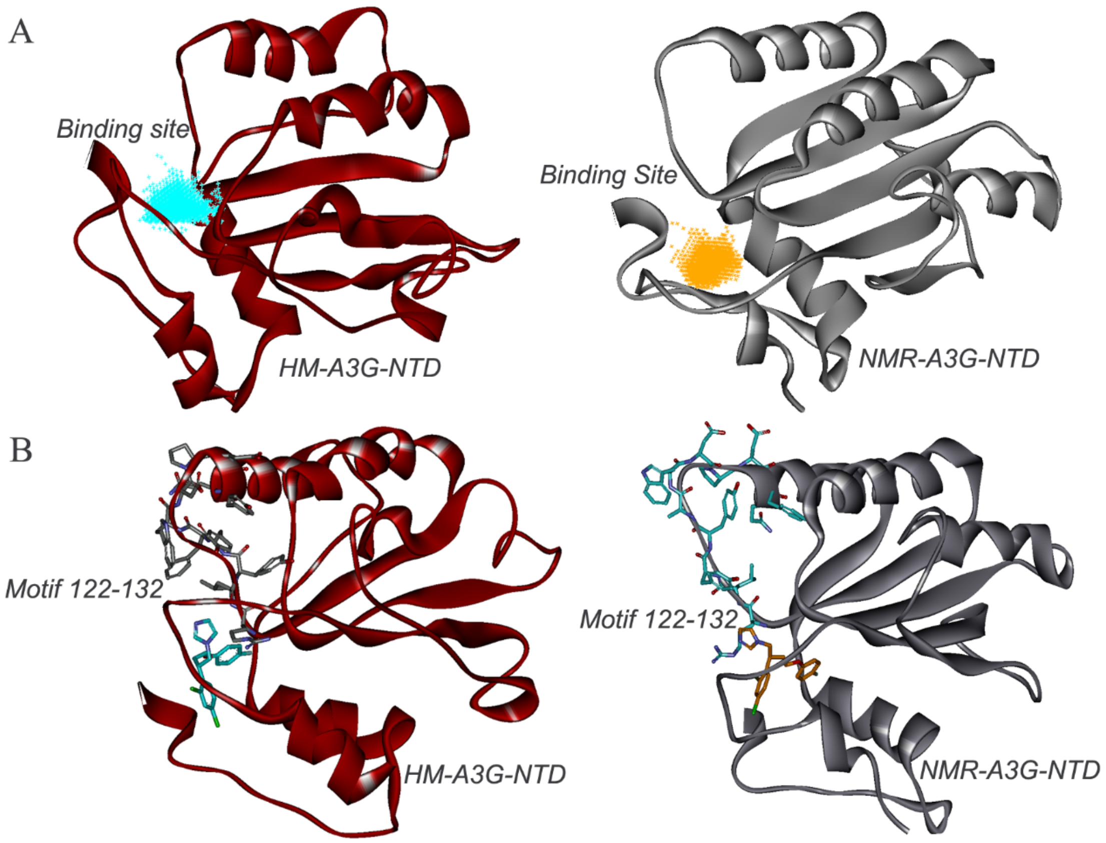

Figure S5. The predicted binding sites in A) the homology structure of A3G-NTD (HM-A3G-NTD) and the NMR structure of A3G-NTD (NMR-A3G-NTD) is closed to A3G/Vif interface (Motif 122-132); B) The similar predicted binding mode of IMB-301 in the binding site for the homology structure of A3G-NTD (HM-A3G-NTD) and the NMR structure of A3G-NTD (NMR-A3G-NTD), respectively.

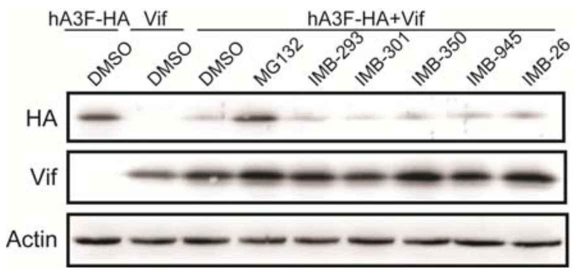

Figure S6. IMB-293, IMB-301, IMB-350, and IMB-945 specifically rescue hA3G degradation rather than hA3F in presence of Vif. 293T cells were co-transfected with hA3F-HA and Vif, 12 hours post-transfection, and the compounds were added at the concentration of 10μM. After 48 hours, cell lysates were harvested and proteins were detected by WB.

Table S1. The similarity analysis between IMB-301 and other active compounds. The similarity was calculated using Open Babel.

Name	Structure	Similarity
**IMB-301**	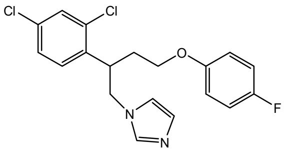	/
IMB-26	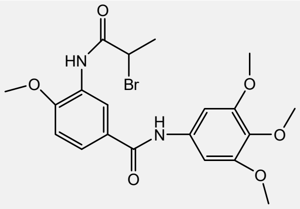	0.22
IMB-35	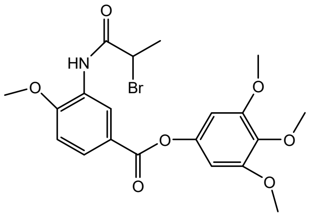	0.23
ZB-MA-1	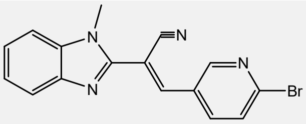	0.20
MM-1	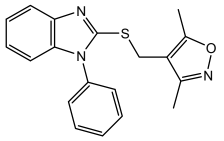	0.24
MM-2	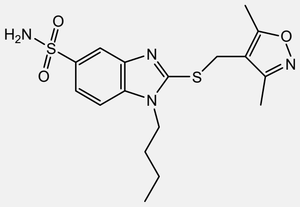	0.23
RN-18	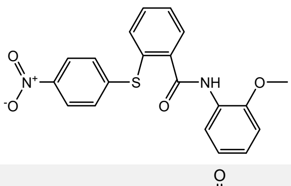	0.20
VEC-5	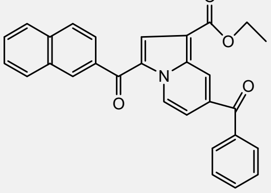	0.20
